# Advanced fabrication techniques for polymer–metal nanocomposite films: state-of-the-art innovations in energy and electronic applications

**DOI:** 10.1039/d4sc04600e

**Published:** 2024-12-18

**Authors:** Muhammad Tayyab, Liu Zizhe, Sajid Rauf, Zixuan Xu, R. U. R. Sagar, Faisal Faiz, Zuhra Tayyab, Rashid Ur Rehman, Muhammad Imran, Anjam Waheed, Rida Javed, A. Surulinathan, Zulakha Zafar, Xian-Zhu Fu, Jing-Li Luo

**Affiliations:** a College of Materials Science and Engineering, Shenzhen University Shenzhen 518055 China; b Guangdong Provincial Key Laboratory of New Energy Materials Service Safety China xz.fu@szu.edu.cn jingli.luo@ualberta.ca jll@szu.edu.cn; c College of Mechatronics and Control Engineering, Shenzhen University Shenzhen 518000 China; d Institute for Frontier Materials, Deakin University Waurn Ponds Victoria Australia; e College of Electronic and Information Engineering, Shenzhen University Shenzhen China; f Graduate School of Engineering, Tottori University Tottori Japan; g School of Civil and Environmental Engineering, Nanyang Technological University 639798 Singapore

## Abstract

Polymer–metal nanocomposites are a fascinating class of materials that synergize the distinct properties of polymers and metals. Incorporating metal nanofillers into polymer matrices significantly enhances electrical conductivity, mechanical strength, and thermal stability through intricate chemical interactions. This review provides an in-depth understanding of current and emerging fabrication techniques for polymer–metal nanocomposite films, with a particular focus on advanced chemical mechanisms and the resulting material properties. By highlighting state-of-the-art innovations, this review distinguishes itself from the existing literature and explores the potential of these nanocomposites in advanced technological applications.

## Abstract 2

Fabrication techniques for polymer–metal composite films in energy and electronic applications: a comprehensive review.

## Graphical abstract

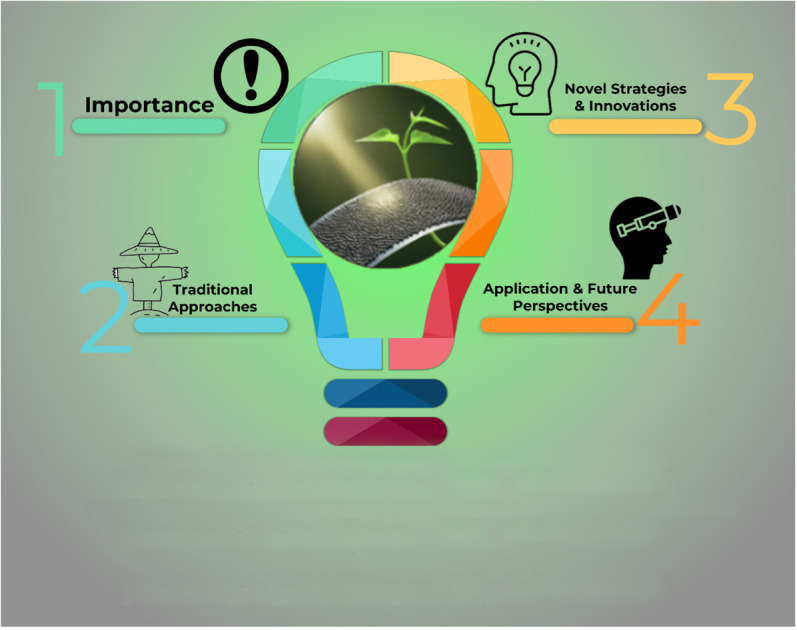

## 1. Introduction

Scientists from a wide range of disciplines have long been interested in metal nanocomposites because of their rich history and versatile qualities.^1–3^ These nanocomposites have shown amazing electrical, magnetic, optical,^4^ and catalytic characteristics,^5,6^ frequently in sharp contrast to their bulk counterparts, in applications ranging from traditional medicine to contemporary electronics.^7,8^ An era of discovery and innovation was ushered in by Faraday's guided synthesis of “divided gold”, which occurred in 1857 and marked a crucial milestone in the systematic creation of nanomaterials.^9,10^ Even in the modern era, metal nanocomposites are still essential to the creation of sophisticated materials for a wide range of uses, including projects involving energy.^11,12^ Many techniques have been developed as scientists go deeper into the creation of polymer–metal nanocomposite thin films for energy applications; each has its own advantages and disadvantages.^13,14^ These production approaches provide fine control over the dispersion and integration of nano-particles,^15^ ranging from casting premade nano-particles to *in situ* creation inside the polymer matrix. Furthermore, improvements in characterization methods allow scientists to clarify the morphological, structural, and functional characteristics of these nanocomposite films, opening the door to customized responses to urgent energy problems.^16–18^

Polymer–metal nanocomposites are gaining significant attention due to their unique combination of chemical and physical properties. These materials leverage the distinct chemical interactions between polymer matrices and metal nano-particles, resulting in enhanced electrical conductivity,^19^ mechanical strength,^20^ and thermal stability. Understanding the underlying chemical processes is crucial for optimizing their performance in various technological applications.^21–23^ Conducting and insulating polymers are essential hosts for metal nanocomposite organization and stabilization in thin-film architecture.^24–26^ These polymers influence the optical, mechanical, electrical, and thermal properties of the resultant nanocomposites while facilitating the integration of metal nano-particles through careful selection and design.^7,27,28^

The potential for polymer–metal nanocomposite films give us oppurtunity to rethink regarding the limitations of energy and electronics applications^20^ to make them an intriguing new frontier.^29,30^ This comprehensive overview delves into the complex world of polymer–metal nanocomposites, revealing their enormous promise in the fields of energy harvesting and electrical devices and exploring the advanced production processes that drive their development.^31–33^ Polymer–metal nanocomposite films are attractive because of their excellent combination of mechanical robustness,^34^ electrical conductivity,^19^ and thermal stability.^27^ These diverse qualities make them extremely desirable for a wide range of applications, from sophisticated energy storage systems to flexible electronics.^35,36^ In a voyage of discovery with this review, we dissect the advanced processes like chemical deposition, physical vapor deposition, and electrospinning/electrospraying that support their synthesis and engineering.

Furthermore, a recurring subject in this investigation is sustainability. It is impossible to overestimate the importance of sustainable manufacturing processes in a time when moving toward greener technology is pressingly necessary.^37^ We thus explore the complexities of attaining sustainability in the process of fabricating polymer–metal nanocomposite films, illuminating the opportunities and inherent difficulties that arise from the convergence of environmental stewardship and materials science.^38,39^ The production of polymer–metal nanocomposite films poses a variety of opportunities and problems in the pursuit of sustainability. Key goals include minimizing the use of resources, cutting down on waste production, increasing energy efficiency,^40,41^ and minimizing the adverse effects on the environment. We hope to clear the path for a more sustainable future in the field of polymer–metal nanocomposite materials by utilizing cutting-edge recycling techniques, investigating renewable resources, and incorporating green chemistry ideas.^42–44^

The revolutionary potential of polymer–metal nanocomposite films becomes more evident as we delve deeper into the rich tapestry of research on them. These materials have the potential to open doors to a future full of scientific advancement and environmentally friendly energy sources. This review article serves as a roadmap for future research projects in the dynamic field of polymer–metal nanocomposite films by offering a thorough analysis of the state of the field, shedding light on both the challenges that need to be addressed and the new fabrication methodologies that are emerging. The multidimensional nature of polymer–metal nanocomposite films is demonstrated by this review paper, which captures the nuances of their manufacturing, characteristics, and potential uses. This review provides researchers and engineers with a road map for navigating the fascinating frontier of polymer–metal nanocomposite materials with purpose and ingenuity by exploring the subtleties of manufacturing techniques, promoting sustainability, and envisioning their transformative impact on energy and electronics.

## 2. Polymer–metal nanocomposite films: overview and properties

### 2.1. Definition and characteristics of polymer–metal nanocomposite films

A class of materials known as polymer–metal nanocomposite films combines metal fillers with a polymeric matrix to produce a special combination of properties. These nanocomposite films have superior mechanical qualities, increased electrical conductivity, and greater thermal stability, among other positive traits. The metal fillers add strength and conductivity, while the polymer matrix offers flexibility. The collective traits of these films are predominantly influenced by the interplay between the metal and polymer components.^45–47^

### 2.2. Advantages and limitations of polymer–metal nanocomposites in energy and electronic applications

The remarkable attributes and extensive array of potential applications of polymer–metal nanocomposite films have sparked significant interest within the energy and electronics domains.^28,48^ High electrical conductivity, adjustable mechanical characteristics, and suitability for flexible substrates are only a few benefits of these nanocomposites. Polymer–metal nanocomposites are used in solar cells, batteries, and supercapacitors in energy applications, allowing for better energy conversion and storage capacities.^49,50^ These films are utilized in electronic circuits, sensors, and flexible displays, offering superior electrical performance and being compatible with newer technology. Polymer–metal nanocomposites do, however, have some drawbacks.^51,52^ A primary obstacle is achieving a constant dispersion of metal fillers in the polymer matrix since an uneven dispersion might result in variable characteristics and decreased performance. Another important element affecting the overall characteristics of the nanocomposite films is the interfacial connection between the metal and polymer phases. Weak interfaces and compromised mechanical strength, load transmission, and electrical conductivity can be caused by inadequate bonding.^53,54^ Furthermore, for successful deployment in applications in reality, issues pertaining to the integrity and long-term stability of polymer–metal nanocomposite films under demanding operating conditions also require attention.

### 2.3. Importance of tailoring properties through nanocomposite film fabrication

A significant factor influencing the properties of polymer–metal nanocomposite films is their fabrication process. The qualities of the nanocomposite films are impacted by the choice of production method, which also affects the interfacial bonding, metal filler dispersion, and overall shape of the films.^55*b*,56^ The ability to precisely regulate the fabrication process enables the modification of metal composition, morphology, and film thickness, resulting in attributes that are specifically designed for intended uses. Different manufacturing techniques, including chemical deposition, electrochemical procedures, and physical vapor deposition, can be used to regulate the film's shape, maximize interfacial bonding, and achieve controlled dispersion of metal fillers.^57^ These techniques can be used to produce polymer–metal nanocomposite films that have appropriate levels of mechanical strength, stability at high temperatures, and conductivity of electricity. Moreover, the development of optimum polymer–metal nanocomposite films is guided by the comprehension of structure–property correlations, which is facilitated by the selection of suitable processing settings and characterization methodologies.

**Fig. 1:**
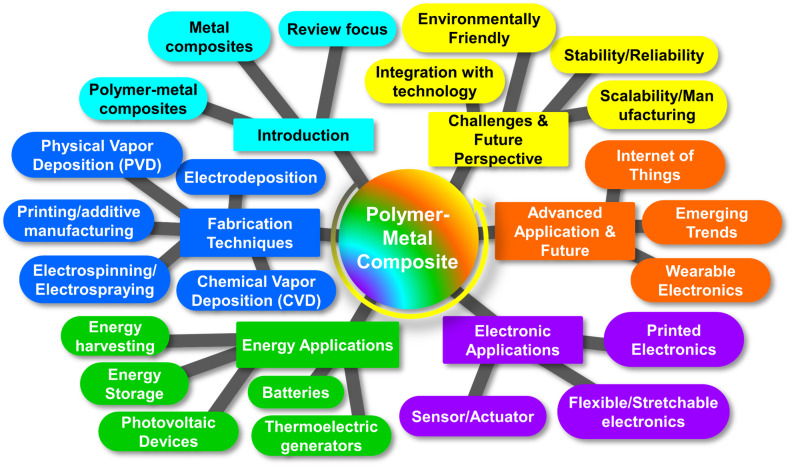
Unveiling fabrication methods for polymer–metal nanocomposites: applications, challenges, and prospects. This figure is adapted from Ceramics International (2024), with modifications in text to suit the current study.^55*a*^

Moreover, Fig. 1 explores fabrication techniques for polymer–metal nanocomposites, materials that blend the strengths of polymers and metals. These nanocomposites hold promise for applications in energy harvesting, storage, and electronics. They can be created through methods like physical vapor deposition, electrospinning, and 3D printing. However, challenges persist in integrating them with current technologies and guaranteeing their stability. In addition, the development of polymer–metal nanocomposite films offers the opportunities to alter their properties to meet the needs of energy and electronics-related applications,^58,59^ The constraints of these nanocomposites can be addressed and the required control over their properties can be achieved with careful selection and optimization of fabrication procedures.^58,60^ A thorough understanding of the techniques used in crafting, as well as their advantages, limitations, and effects on the properties of films, is crucial to the development of excellent polymer–metal nanocomposite films that are suitable for a variety of applications. Here are some polymer–metal nanocomposite film fabrication techniques.

## 3. Fabrication techniques for polymer–metal nanocomposite films

### 3.1. Electrodeposition

Electrodeposition, also known as electroplating or galvanization, involves utilizing an electric current to coat a conductive object with a layer of metal. In this process, the metal substance to be deposited is dissolved into a solution to form the anode, while the object to be coated acts as the cathode,^12,61^ When an electric current flows through the solution, metal ions migrate from the anode to the cathode. They go through reduction there, leaving behind a uniformly thin coating of metal on the object's surface. Electrodeposition has widespread applications in providing corrosion resistance and producing electronic components, conductivity enhancement, and decoration.^62,63^

**Fig. 2:**
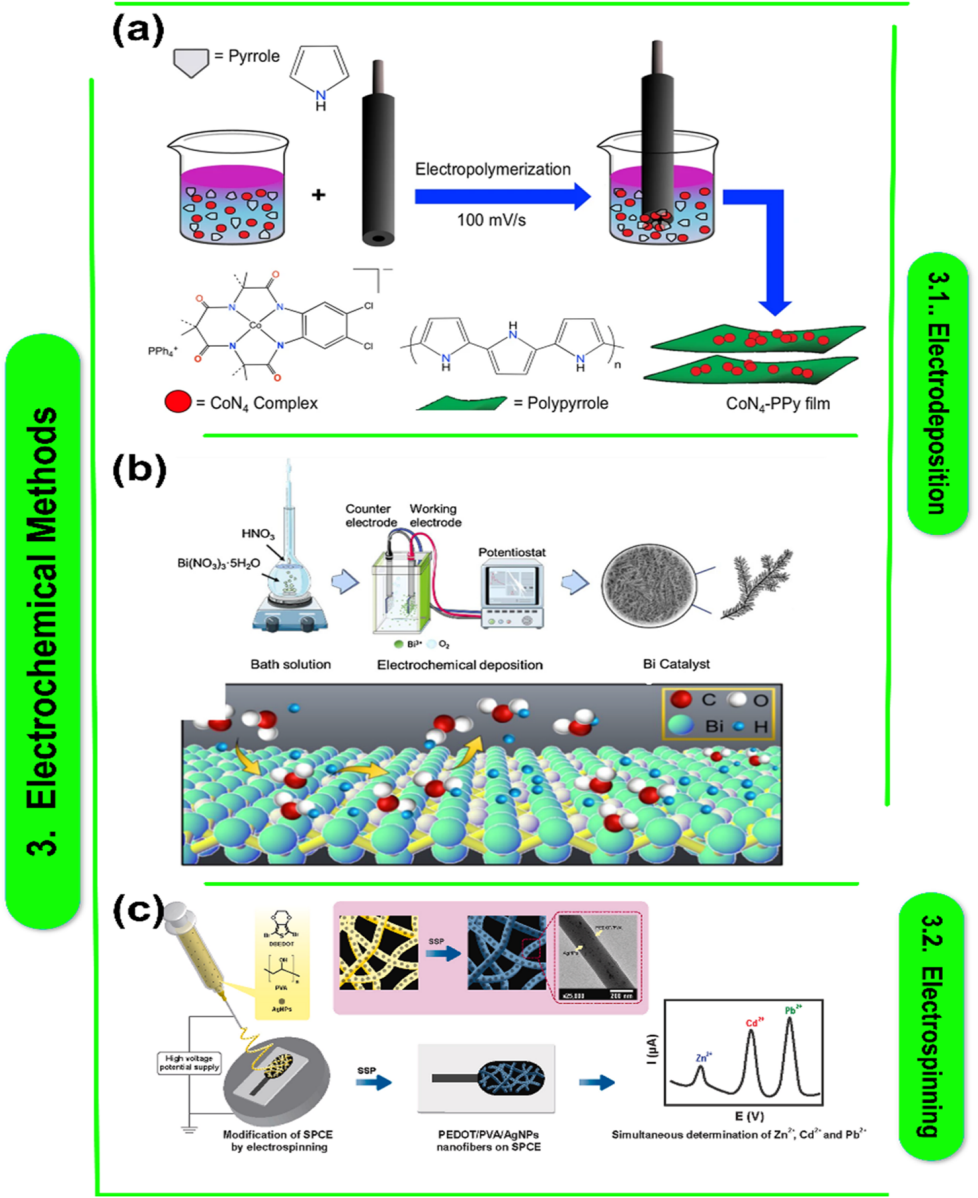
(a) Diagram showing the electrochemical deposition of PPy sheets using the pyrrole and CoN_4_ combination. An electrode made of glassy carbon (GCE) was used for the deposition process. The Co metal's elemental composition, bond formation, and oxidation state in the thin film were examined using X-ray photoelectron spectroscopy (XPS).^65^ This figure has been reproduced from ref. 65 with permission from Nature Publishing Group, copyright 2019. (b) Diagram of the constant current two-electrode bismuth electrodeposition device.^66^ This figure has been reproduced from ref. 66 with permission from Nature Publishing Group, copyright 2022. (c) Diagrammatic illustration of solid-state polymerized poly(3,4-ethylenedioxythiophene) conductivity-based electrospun nanocomposite fibers that enable simultaneous electrochemical metal ion detection.^68^ This figure has been reproduced from ref. 68 with permission from Elsevier, copyright 2022.

C. M. Parnell *et al.* claim that electrochemical oxidation accomplished by electrochemical deposition may effectively produce PPy films employing a cobalt salt complex (Co(OAc)_2_) and CoN_4_. Opting for an acidic medium over organic or alkaline solvents proved to be the optimal electrolyte choice. Unlike Co(OAc)_2_,^64^ (Fig. 2a) Charlette's research elucidated the distinct redox solvability and properties of CoN_4_, facilitating a higher accumulation of CoN_4_ beneath PPy sheets and a consequent rise in total capacitance. CoN_4_-PPy showed the largest specific capacitance at 5 mV s^−1^, reaching 721.9 F g^−1^ in 0.1 M HClO_4_. Moreover, exceptional electrochemical stability was seen under acidic conditions for up to 1000 cycles. Better capacitance and charge storage were demonstrated by the GCD's low charging or discharging rate. The CoN_4_-PPy compound demonstrated remarkable stability, retaining a good specific capacitance retention even after 500 cycles of charge and discharge. These films' incredibly accurate capacitance and ease of manufacturing make them ideal as supercapacitor electrode materials.

According to Parnell *et al.* current density and deposition duration are regulated in galvanostatic deposition to regulate catalyst deposition thickness and shape while maintaining a constant current.^65^Fig. 2b shows a schematic representation of the controlled current two-electrode configuration used for the one-step fabrication of a nanocrystalline bismuth electrode. In a solution containing 10 mM Bi^3+^ in 0.2 M NO_3_, they used polycrystalline copper foil as the primary electrode and stainless steel as the counter electrode. By adjusting the deposition period, the catalyst's shape and deposition thickness were adjusted at 15 mA cm^−2^. After 500 seconds of deposition, nano-Bi branches first developed on the matrix's surface, then at 800 and 1200 seconds, acute spiniform secondary structures appeared. A 1500 s deposition period resulted in the production of bi-agglomerated films. The branches merged to form grains as a result.^67^

Several studies have investigated the fabrication and characteristics of polymer–metal nanocomposite films for energy-related purposes. Ko *et al.*^69^ demonstrated the simplicity of producing electrically conductive polypyrrole–magnetic metal nanocomposite films through electrodeposition, offering the prospects for tailored magnetism. By using MXene, a 2D transition metal carbide, to speed up the electrochemical polymerization of conjugated polymer–MXene nanocomposite films and produce high-performing microsupercapacitors, Qin *et al.*^70^ made significant progress in this field. Meng *et al.*^71^ and Liu *et al.*^72^ focused on improving these films' electrochemical characteristics. Meng *et al.* used a novel method to create flexible carbon nanotube/polyaniline paper-like films with superior electrochemical properties, whereas Liu *et al.*^73^ accomplished this by combining polyoxometalate with poly(3,4-ethylenedioxythiophene) (Fig. 2c) to improve electrochromic performance. These investigations collectively underscore the potential of polymer–metal nanocomposite films for diverse energy applications. Table 1 presents a collection of research papers focusing on various polymer–metal nanocomposite materials for energy-related applications. Each entry provides a concise abstract summary, details on the specific metal and polymer nanocomposites used in the study, along with proposed research questions and future directions. Topics range from electrodeposition techniques to flexible supercapacitors and electrochromic films. These papers highlight the versatility of polymer–metal nanocomposites in addressing key challenges in energy storage and conversion, offering insights into novel synthesis methods and potential applications across different fields.

**Table 1:** Summary of research papers on polymer–metal nanocomposite materials for energy applications

Article title	Abstract summary	Metal & polymer nanocomposite used	Future research	Ref.
Electrodeposited conducting polymer–magnetic metal nanocomposite films	Electrodeposition makes it easy to create nanocomposite films of magnetic metals and conductive polymers	Polypyrrole (PPy) and NiFe alloy	Is it possible to achieve tailored magnetism by simplifying the electrodeposition process of conductive polymer and magnetic metal nanocomposites?	69
Polymer–MXene nanocomposite films formed by MXene-facilitated electrochemical polymerization for flexible solid-state microsupercapacitors	The nanocomposite produced using MXene exhibits outstanding electrochemical characteristics, indicating substantial promise for a variety of energy storage applications	MXene and polymer	How can we design a novel MXene-enabled electrochemical polymerization strategy to produce polymer–MXene nanocomposite films with customized properties and enhanced electrochemical performance for a range of energy storage uses?	74
Enhanced electrochromic performance of nanocomposite films by the combination of polyoxometalate with poly(3,4-ethylenedioxythiophene)	The electrochromic performance of the PEDOT film is significantly improved by the addition of P_2_W_18_	Polyoxometalate (POM) and poly(3,4-ethylenedioxythiophene) (PEDOT) clusters K_6_P_2_W_18_O_62_⋯14H_2_O (P_2_W_18_)	What impact does P_2_W_18_ integration have on the visible spectrum light absorption characteristics and electrochromic performance of the PEDOT film?	75
Flexible carbon nanotube/polyaniline paper-like films and their enhanced electrochemical properties	These nanocomposite electrodes, which resemble paper, have potential uses in new kinds of energy storage devices	Carbon nanotubes (CNTs) and polyaniline (PANI)	How can we develop flexible and high-performance electrodes for energy storage devices using CNTs/PANI nanocomposites?	76
Electrochemical deposition of polypyrrole nanostructures for energy applications: a review	Electrochemical techniques for depositing polypyrrole nanostructures and their potential applications are examined	Polypyrrole (PPy)	What is the influence of nanostructuring on the performance and stability of polypyrrole electrodes in energy devices?	77
Electrodeposition of polymer–semiconductor nanocomposite films	When films made from the same solutions are compared, films electrodeposited with nano-particles show better quality and characteristics	CdS or CdTe combined with poly(vinyl acetate-*co*-crotonic acid)	How do hybrid polymer–semiconductor nanostructured films made by co-electrodeposition differ from films made by other techniques in terms of their attributes and qualities?	78
Electropolymerized polyaniline stabilized tungsten oxide nanocomposite films: electrochromic behavior and electrochemical energy storage	Dual electrochromism is seen in the nanocomposite films at both positive and negative potentials	Polyaniline (PANI) and tungsten oxide (WO_3_)	What characteristics of PANI/WO_3_ nanocomposite films make them suitable for use in energy storage and electrochromic devices?	79

According to M. H. Naveen *et al.*^80^ CP-carbon nanocomposites are a potential option because of their distinct features, especially in electrochemical devices. There have been several documented techniques for creating new CP nanocomposite materials, including chemical-based transformation, vapor polymerization process, template-oriented production, *in situ* CP nanocomposite synthesis, *etc.* In addition to these approaches, electro-chemical processes offer a practical way to create CP nanocomposites, and they make it simple to regulate the nanocomposites' conductivity, morphology, thickness, and chemical state. Nanocomposites consisting of CPs are made up of CPs as the main component and one or more secondary components, which could be any kind of biological, inorganic, or organic species. These substances include molecular species, such as metal phthalocyanines, metal ions, NPs, carbon materials, nanostructures of metal and metal oxides, and physiologically active substances, such as proteins, enzymes, antibodies, and antigens.

### 3.2. Electrospinning/electrospraying

Electrospinning involves the chemical synthesis of polymer solutions and their interaction with metal salts to form nanofibers. The chemical properties of the polymer and metal precursors, such as solubility, reactivity, and intermolecular forces, are critical in determining the quality and functionality of the resulting nanocomposite fibers. In the electrospinning process, a tiny nozzle called a spinneret receives a high voltage between it and a collector. When the polymer solution is fed into the spinneret, it experiences a high electrostatic force that pulls it out into a fine jet. This fine jet then goes through elongation and solidification to produce ultrafine fibers. After that, the fibers are gathered and organized in a non-woven mat structure on the grounded collector.^81^ In contrast, electrospraying is the process of using an electromagnetic field to disperse a liquid suspension or solution into tiny droplets. To obtain solid particles, these droplets are usually gathered on a substrate or let too dry in the atmosphere. Applications like coating deposition, medication delivery, and nanofiller production frequently involve electrospraying.^82^ Electrospinning involves the chemical synthesis of polymer solutions and their interaction with metal salts to form nanofibers. The chemical properties of the polymer and metal precursors, such as solubility, reactivity, and intermolecular forces, are critical in determining the quality and functionality of the resulting nanocomposite fibers.^83^ For example, in the electrospinning of polyaniline (PANI) with gold nano-particles (AuNPs), the gold chloride (AuCl_3_) is reduced *in situ* during the spinning process, leading to the formation of AuNPs within the PANI matrix:3PANI + AuCl_3_ → PANI(Au) + 3HCl

Electrospinning involves the chemical synthesis of polymer solutions and their interaction with metal salts to form nanofibers. The chemical properties of the polymer and metal precursors, such as solubility, reactivity, and intermolecular forces, are critical in determining the quality and functionality of the resulting nanocomposite fibers. The creation of nano-particles has emerged as a major area of research that has generated a lot of interest. Nanomaterials have been prepared using numerous physical (ball milling, vacuum evaporation–condensation, spray pyrolysis, sputtering method, *etc.*) and chemical (template-directed procedures, solution-phase, vapour deposition, hydrolysis, *etc.*) techniques. The development of spinning, a superior physical synthetic technique, has led to a revolution in the manufacturing of sophisticated continuous carbon fibers, and the introduction of electrospinning and electrospraying has brought about easy, affordable, and industrial ways to create nanomaterials. Different 3D spheres, 2D nanofilms, 1D nanofibers, and other novel structures can be created using electrospray and electrospinning. As an illustration, the distinct dimensional impact of the electrospun electrolyte utilized in DSSCs enhances quasi-solid electrolytes, while the uniform application of particles *via* electrospray, as depicted in Fig. 2e, improves the electrochemical characteristics of supercapacitors. Low polarization loss and improved long-term performance are two other benefits of electrospun materials for fuel cells. Additionally, a number of interesting metrices, including pores, hollow, infused, and core shells, for batteries made from lithium-ion and hydrogen vaults with remarkable stability in cycles and capacity for batteries, can be achieved by carefully modifying the production process. These nanomaterial technologies still need to be improved, though, notably in the areas of dimensional homogeneity, scale-up for industrialization, and more exact control over the nanostructure.^82^

Electrospinning has found extensive applications in the production of polymer–metal nanocomposite films for energy-related uses. P. Miao *et al.*^84^ and Zhang *et al.*^85^ emphasize the versatility of this method, allowing for the creation of intricate structures with precise composition and morphology control. The resultant films are suited for use in proton exchange membrane fuel cells and high-performance lithium-ion batteries due to their higher surface area, porosity, and linked pore topologies. J. Yan *et al.*^86^ further showcase the potential of such films by crafting electropolymerized polyaniline stabilized tungsten oxide nanocomposite films, which demonstrate dual electrochromism and electrochemical capacitive behaviors. Z. Lin *et al.*^87^ extend this discussion by exploring the synthesis of inorganic fibers *via* polymer-mediated electrospinning, with a specific focus on their utility in energy harvesting, storage, and sensor applications.

Table 2 presents a summary of various research papers focusing on the utilization of polymer-based nanofibers for energy-related applications. Each paper's abstract is summarized, highlighting the key findings and applications of the respective nanofiber nanocomposites. The table also identifies the specific metal and polymer nanocomposites used in each study, shedding light on the materials employed for these advanced applications. Additionally, the future research directions suggested by each paper are outlined, providing insights into the potential areas of further exploration and development in the field of polymer-based nanofibers for energy and environmental applications.

**Table 2:** Summary of research papers exploring polymer-based nanofibers for energy-related applications, highlighting key findings, materials used, and future research directions

Paper title	Abstract summary	Metal/polymer nanocomposite used	Future research	Ref.
Electropolymerized polyaniline stabilized tungsten oxide nanocomposite films: electrochromic behavior and electrochemical energy storage	The nanocomposite films display dual electrochromism under both positive and negative potentials	Polyaniline (PANI) and tungsten oxide (WO_3_)	Future inquiries could center on enhancing the electrochromic and energy storage attributes of PANI/WO_3_ nanocomposite films by refining synthesis conditions and exploring alternative polymer/inorganic particle combinations	79
Carbonized electrospun polyvinylpyrrolidone/metal hybrid nanofiber nanocomposites for electrochemical applications	Electrospun nanofibers embedded with cobalt nano-particles enhance charge transfer efficiency at the electrode surface	Polyvinylpyrrolidone (PVP) and cobalt chloride	Future research paths might involve using alternative metal precursors to enhance the properties of carbon nanofiber nanocomposites. Additionally, investigating the application of these nanocomposites in diverse fields like energy storage devices, sensors, and catalysis could be pursued	88
Layer-by-layer assembly of MXene and carbon nanotubes on electrospun polymer films for flexible energy storage	The nanocomposite electrodes possess flexibility and foldability	Ti_3_C_2_T_*x*_ (MXene) and polycaprolactone (PCL)	Future research efforts could focus on developing nanocomposite electrodes with improved mechanical properties and exploring their performance in flexible and wearable energy storage devices	89
Coaxial electrospun nanostructures and their applications	Coaxial electrospinning has the potential to fabricate polymer nanofibers with distinctive core-sheath or hollow structures	Metal salts	Future research directions might include developing innovative materials and structures, as well as exploring their potential applications across various fields	90
Polymer-based electrospun nanofibers for biomedical applications	Electrospinning allows for the creation of nanofibers tailored for a variety of applications, including energy, biotechnology, healthcare, and environmental engineering	Copolymer of ε-caprolactam and hexamethylendiaminadipate	Future research could focus on optimizing fabrication methods of electrospun nanofibers for specific biomedical applications, exploring controlled drug delivery capabilities by manipulating nanofiber wall thickness, investigating *in vivo* stability, conducting detailed toxicity studies for clinical approval, and further developing porous 3D scaffolds from bioabsorbable polymers for tissue engineering	91

In addition, electrospinning and electrospraying are key methods for creating nanoscale fibers and particles, respectively, with applications in coating deposition and medication delivery. These methodologies, in conjunction with a range of chemical and physical approaches, enable the synthesis of a broad array of nanomaterials featuring distinctive structures. Despite their potential in enhancing energy storage devices and environmental applications, further improvements are required for scalability and structural control.

### 3.3. Printing and additive manufacturing approaches

#### 3.3.1. Inkjet printing

**Fig. 3:**
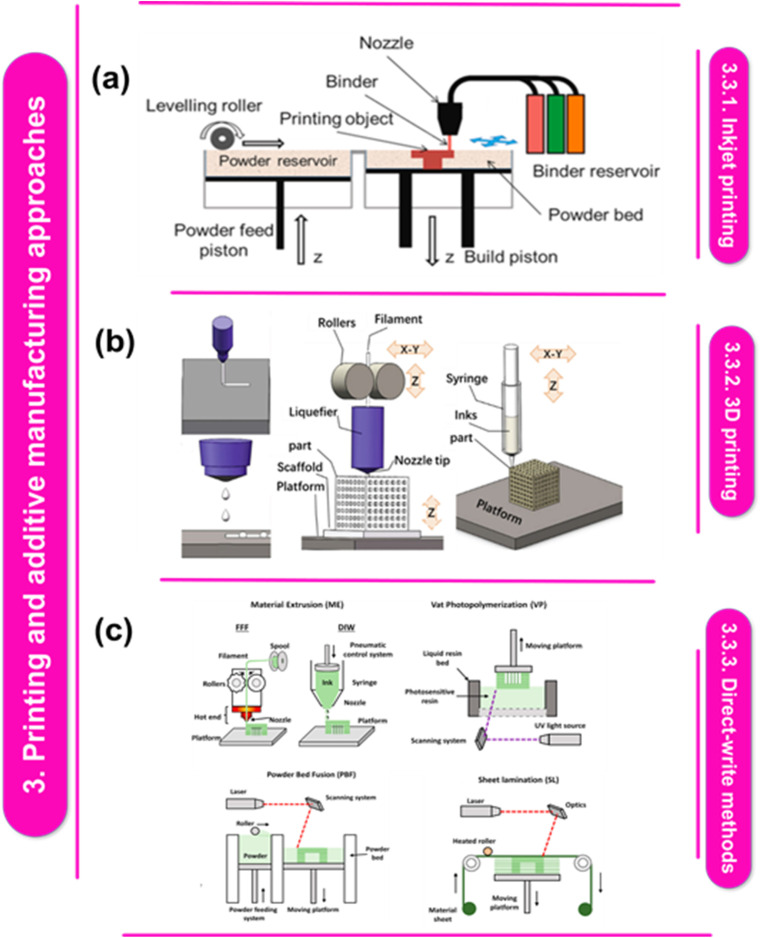
(a) Diagram illustrating IJ and CIJ-DW printing.^92^ This figure has been reproduced from ref. 92 with permission from Chinese Journal of Mechanical Engineering, copyright 2022. (b) The SLS process' schematic, the MJP method, the concepts of powder bed inkjet 3D printing.^100^ This figure has been reproduced from ref. 100 with permission from Elsevier, copyright 2023. (c) Technique diagrams for 3D printing: melting a solid filament, then forcing it through a nozzle and depositing it layer by layer is a 3D printing technique called filament fused fabrication (FFF). Powder bed fusion (PBF) creates crosslinked networks. Vat photopolymerization (VP) uses heat energy to melt powdered material, producing three-dimensional things; material jetting (MJ) selectively deposits build material droplets to construct three-dimensional objects using a process called sheet lamination (SL). Reproduced with permissions.^101^ This figure has been reproduced from ref. 101 with permission from Wiley-VCH Verlag GmbH & Co. KGaA, copyright 2022.

Using ink droplets or functional materials, inkjet printing is a non-contact printing method that imprints patterns on a substrate. It works on the basis of the idea that thermal or piezoelectric techniques can be used to selectively deposit small droplets onto a surface. A print head with several nozzles pours ink onto the substrate in a controlled way during inkjet printing. Because the nozzles are computer-controlled, the droplets may be precisely positioned. For a certain application, the ink or material is formulated with the desired qualities, such as conductivity, dielectric behaviour, or biological compatibility. Fig. 3a shows the schematic representation of the CIJ-DW & IJ printing.^92^ Hydroxyapatite (HAp) stands as a quintessential biomaterial for bone regeneration, owing to its remarkable resemblance in composition and structure to bioapatites present in hard tissues. The creation of porous HAp intended as a scaffold for fostering bone growth and regeneration has presented a significant hurdle for the biomaterial-focused scientific community. However, a new era of manufacturing processes has dawned with the advent of additive manufacturing technologies, offering the advantages of swift, precise, controlled, and potentially scalable fabrication methods. These technological strides have unveiled fresh avenues in the domain of bioceramic scaffolds.^93^

In fact, there are three main types of 3D printing, which is often referred to as bioprinting: microextrusion printing, laser-assisted printing, and inkjet printing. While all three use comparable spatial strategies, each one discharges ink differently. Various techniques, including thermal, acoustic, or microvalve mechanisms, are used in inkjet printing to precisely dispense a liquid in predetermined volumes onto material sites. In laser-assisted printing, the forward-induced laser method is employed. Drop by drop, a high-pressure bubble generated by a laser pulse delivered to an absorbent layer propels biomaterial inks onto the printing surface. Microextrusion printing is a robotic system whose dispensing nozzles are powered by mechanical (piston or screw) or pneumatic (air or compressed gas). It creates continuous ink layers or filaments rather than discrete drops. Regarding the field of bioceramic 3D printing, hydroxyapatite (HAp) has improved mechanical characteristics and osteogenic potential *in vitro* and *in vivo*. A range of studies have explored the potential of inkjet printing for creating polymer–metal nanocomposite films for energy applications. Burlikowski *et al.*^94^ proposed a novel nanocomposite material for 3D printing, which demonstrated superior magnetic properties compared to traditional materials. Kim *et al.*^95^ successfully fabricated a multifunctional nanocomposite laminate for energy harvesting and storage using inkjet-printed electrodes. Zhao *et al.*^96^ introduced a room-temperature metal printing technique, which could potentially be integrated with inkjet printing to create high-performance polymer–metal nanocomposites. Finally, Lesch *et al.* highlighted the use of inkjet printing for the fabrication of catalyst layers in electrochemical energy conversion, suggesting a potential application in the development of polymer–metal nanocomposite films.^97^

Table 3 summarizes key findings regarding polymer–metal nanocomposite materials utilized in advanced printing technologies. The initial entry outlines the introduction of an innovative metal printing method at room temperature, employing polymer-assisted photochemical deposition (PPD), highlighting its effectiveness in producing continuous, smooth, and reflective metal films. The second entry focuses on the development of a conductive nanocomposite for inkjet printing applications, emphasizing its adhesion, resistivity, and compatibility with MEMS processes. Both entries underscore the potential research gaps and future directions for optimizing these nanocomposite materials in numerous applications.

**Table 3:** Polymer–metal nanocomposite materials in advanced printing technologies

Article title	Main finding	Polymer used	Metal used	Research gaps	Ref.
Printing continuous metal structures *via* polymer-assisted photochemical deposition	The present limits of additive manufacturing are overcome by the new room-temperature metal printing technology that uses polymer-assisted photochemical deposition (PPD). Its effectiveness is highlighted by the fact that the resultant metal films are smooth, continuous, and reflectivity-matched with those that are vacuum-deposited	Photochemical deposition with polymer assistance (PPD)	Silver, gold, and platinum	Exploring other metal precursors and polymer matrices to extend the range of printable materials, adapting the PPD process for printing other functional materials like semiconductors and dielectrics for electronics and energy storage applications	96
Carbon nanotubes–SU8 nanocomposite for flexible conductive inkjet printable applications	Development of a conducting nanocomposite for inkjet printing with good adhesion, resistivity, and compatibility with MEMS processes	Epoxy resin novolac bisphenol A (SU8)	Multi-walled carbon nanotubes	Optimization of the nanocomposite material for specific applications such as flexible and transparent electrodes for touch screens, organic photovoltaic cells, and flexible displays	98

This in-depth examination focuses on the nanocomposite structures of 3D printed HAp scaffolds, wherein the calcium phosphate phase enhances mechanical qualities and provides special features by collaborating with other ceramics or polymers. The review outlines and takes into consideration all of the many applications of these 3D-printed HAp scaffolds in the area of bone tissue engineering, emphasizing their main purposes. It also clarifies the most recent advancements in the multifaceted HAp-based systems, which combine a variety of attributes to enable cutting-edge treatments like angiogenesis, antimicrobial activities, bone regeneration, and even cancer treatment. The reader is now prepared to begin this academic journey, in which the benefits of HAp-based scaffolds are emphasized, their potential is acknowledged, and the fields of advanced therapy beckon with promises of advancement and hope. Many industries use inkjet printing, including additive manufacturing, printed electronics, bioprinting, and graphics and textile printing. High resolution, scalability, personalization, and the capacity to print on a variety of substrates are some of its benefits. For intricate pattern manufacturing, low-volume production, and prototyping, inkjet printing is especially helpful.^93^

There are four main components of Direct Writing (DW) technologies are the 3D stage, deposition nozzle, ink reservoir, and energy supply (Fig. 3a). In continuous inkjet direct writing (CIJ-DW), a steady stream of binder is expelled through a pointed tip. Because of the Plateau–Rayleigh flow instability phenomena, the binder divides into droplets when it departs from the nozzle. The split droplets are either directed to the specified deposition area or collected for later use with the aid of an electric field. It's essential to distinguish between conventional Inkjet (IJ) printing and CIJ-DW techniques. In IJ printing, a continuous stream of binder jets is sprayed onto the powder bed to solidify it. CIJ-DW offers versatility in deposition onto various substrates, such as polydimethylsiloxane (PDMS), textiles, and polyethylene terephthalate films, providing a notable advantage. Because of its adaptability, CIJ-DW can be applied to a wide range of materials, including metallic pigments, polymers, graphene nano-particles, and carbon nanotubes. Therefore, the method is perfect for improving the properties of textiles and conducting electronic printing. According to A. Zhakeyev *et al.*, the American Society for Testing and Materials (ASTM F2792-12a) outlines over fifty different AM technologies, categorized into six basic processes: sheet lamination, material extrusion, binder jetting, substance jetting, powder bed fusion, and vat photopolymerization. Grouping these technologies based on the physical states of the raw materials (solid, powder, or liquid) and the mode of fusing them (heat, ultraviolet (UV) laser, radiation, or electron beam) provides a simpler way to differentiate between them.^102^ Examples include fused deposition modeling (FDM), powder bed inkjet (Fig. 3b) 3D printing (inkjet 3D), stereolithography (SLA), laminated object manufacturing (LOM) and direct ink writing (DIW). Liquid photosensitive resin serves as a useful feedstock for various AM processes creating polymer structures, such as SLA and inkjet printing. In a specific study, an ExOne R2 inkjet 3D printer was utilized to create models using a mixture of copper particles combined with a standard binder. The results demonstrated high density and purity after sintering, with significant improvements observed in overhangs treated with nano-particles.

#### 3.3.2. 3D printing

3D printing, or additive manufacturing, has been more well-known in recent years as a revolutionary technology that has the power to fundamentally change a variety of industries. A revolutionary step forward in materials engineering,^99^ the combination of metals' excellent mechanical qualities and polymers' adaptability is provided by the 3D printing of metal and polymer nanocomposites. This revolutionary approach focuses research and development in the sector because it not only presents a novel class of materials with configurable features but also broadens the breadth of applications. This long-standing contradiction is addressed by the development of metal–polymer nanocomposites, which are made possible by 3D printing technologies and offer a platform for the precise and effective integration of metal and polymer components.^110^ The comprehensive schematic of 3D printing methods is displayed in Fig. 3c. Various 3D printing technologies have been studied for producing polymer–metal nanocomposite films. Mazeeva *et al.*^103^ (2023) offer an overview, including direct ink writing (DIW), stereolithography (SLA), filament-fused deposition modeling (FDM), and binder jetting (BJ). Ryder *et al.*^104^ (2018) specifically explore FDM's use in ABS-SS nanocomposite fabrication, demonstrating its viability. Wang *et al.*^105^ (2017) and Dickson *et al.*^106^ (2020) highlight 3D printing's potential for high-performance polymer nanocomposites, with Wang noting precision and cost-effectiveness and Dickson emphasizing improved mechanical properties in fiber-reinforced polymer nanocomposites. These studies collectively affirm 3D printing's promise for polymer–metal nanocomposite film fabrication, with FDM being particularly noteworthy.

Furthermore, Table 4 summarizes various research papers focusing on different aspects of 3D printing technologies for the fabrication of polymer–metal nanocomposites and related materials. Each entry provides a brief overview of the research paper, including the materials studied, the specific 3D printing techniques employed, and the identified research gaps. These gaps primarily revolve around the need to explore alternative magnetic materials beyond Nd–Fe–B and permalloys, understand the capabilities and limitations of polymer additive technologies, develop printable polymer nanocomposites with high performance, address limitations in 3D printing processes, investigate scalability and practical applications of larger-scale 3D structures, and explore the impact of factors such as fiber content, orientation, and length in fiber-reinforced thermoplastic nanocomposites. Overall, the table highlights the ongoing efforts to advance 3D printing techniques and materials for the fabrication of polymer–metal nanocomposites and related products, while also identifying areas for further research and development.

**Table 4:** Exploring research gaps and advancements in 3D printing technologies for polymer–metal nanocomposites

Article title	Summary	Polymer–metal nanocomposite	Research gaps	Challenges	Potential solutions	Ref.
3D Printing technologies for fabrication of magnetic materials	Research on polymer 3D printing technologies for magnetic materials highlights gaps in investigating magnetic materials beyond Nd–Fe–B and permalloys and understanding polymer additive techniques	PLA and carbonyl iron	Need for research on other magnetic materials – understanding capabilities and limitations of polymer additive technologies	Limited exploration of magnetic materials beyond Nd–Fe–B and permalloys	Expanding research into alternative magnetic materials and enhancing polymer additive technology knowledge	103
Additive manufacturing of polymer matrix nanocomposite materials with aligned or organized filler material: a review	3D Printing has benefits for nanocomposite fabrication, but gaps remain. There's a need for high-performance printable polymer nanocomposites to overcome 3D printing limitations	Polymer nanocomposite reinforced with metal particles/fibers	Need for printable polymer nanocomposites – addressing limitations in 3D printing	Limitations in the performance of printable polymer nanocomposites	Development of high-performance printable polymer nanocomposites	107
3D Printing of mixed matrix films based on metal–organic frameworks and thermoplastic polyamide 12 by selective laser sintering for water applications	MMF effective adsorbents, exploration of other MOFs and scalability needed	Thermoplastic polyamide 12, various metal–organic frameworks	Investigating the scalability and practical applications of larger-scale 3D structures	Scalability and practical application challenges of larger-scale 3D structures	Research into scalable production methods and practical application testing	108
Polymer nanocomposite manufacturing by FDM 3D printing technology	FDM for 3D element creation, issues with printing and exploring single polymer nanocomposites	Polymer–metal nanocomposite	Issues related to intermittent printing – exploring single polymer nanocomposites	Intermittent printing issues with FDM technology	Improvement of FDM technology for consistent printing and exploration of single polymer nanocomposites	109

According to S. Park *et al.*^111^ additive manufacturing, or 3D printing, has made it possible to fabricate items on demand with intricate mechanical and electrical capabilities and geometries. AM technologies have been developing in a range of industrial and new applications, and they often involve polymers and nanocomposites. Notwithstanding recent advancements in the 3D printing of polymer nanocomposites, a number of obstacles still need to be overcome before additively created polymer nanocomposites are widely used. These obstacles include the subpar quality of manufactured goods and the scarcity of materials for 3D printing. It is well known that polymers and polymer nanocomposites can be used to build three-dimensional objects through 3D printing. While there is still much to learn about many 3D printing processes, there are a few that are well-established, such as material extrusion (ME), VP (or material jetting MJ), binder jetting (BJ), stereolithography, and PBF. There are distinct advantages and challenges associated with each 3D printing technique used to create polymer nanocomposites. Among these are the requirements for specific polymer properties, like shape, condition (liquid or solid), and physical characteristics (melting point and viscosity). As such, while selecting 3D printing processes (geometry complexity, including resolution and mechanical characteristics), a user must consider application requirements, materials, and cost.

##### 3.3.2.1. *In situ* extrusion of materials using a polymer matrix and dry carbon fiber

*In situ* impregnation has been applied to CFRPCs using material extrusion-based 3D printing. This method involved feeding reinforcing fibers and other sources of thermoplastic resin filament into the printer head. The polymer filament was melted by the printing head's heating element, impregnating the reinforcing fibers. Following extrusion, these fibers hardened and fused together on the hot table. Continuous carbon fiber reinforced PLA demonstrated a tensile modulus of 19.5 ± 2.08 GPa and a strength of 185.2 ± 24.6 MPa, which are much higher than those of pure PLA. After 3D printing PLA with a 6.6% fiber volume percentage, CF/PLA nanocomposites with an average flexural strength of 335 MPa and flexural modulus of 30 GPa were produced. With 1K fiber tow and a 0.3 mm nozzle output diameter, the printed coupon showed a 50% increase in fiber volume percentage, resulting in a longitudinal tensile modulus of 81.0 ± 3.1 GPa and a strength of 731 ± 32 MPa. In 3D printing, control over the impregnation process is essential, even if the *in situ* impregnation method allows for free selection of the resin, fiber, and fiber tow size.^112^

#### 3.3.3. Direct-write methods

A class of additive manufacturing techniques known as “direct-write methods” enables the exact deposition of materials through a pen- or nozzle-like device. Using these techniques, micron-scale designs or structures can be printed directly onto substrates. A variety of technologies fall under the category of “direct-write methods,” such as the previously discussed aerosol jet printing, inkjet printing, extrusion-based printing, and laser-induced forward transfer (LIFT). The materials that can be printed with these methods are diverse and can include living cells, inks, polymers, and nano-particles. Applications for direct-write technologies can be found in bio fabrication, sensors, electronics, and microelectronics. With perfect control over the deposition process, they make it possible to create functional devices, interconnects, circuits, and even complex biological structures. These techniques enable the fabrication of complicated biological structures, functional devices, circuits, and interconnects by enabling the exact deposition of materials. For research, prototyping, and production applications, direct-write technologies are useful because they can print on non-planar surfaces and combine various materials.^93,113^

Using ink-type feedstocks, one of the most widely used AM techniques is called direct ink writing (DIW). This recently developed method now encompasses all types of ceramic materials, including concrete, nanocomposites, bio-ceramics, and alkali-activated materials. The term “DIW” describes a broad range of extrusion-based additive manufacturing (AM) technologies that are utilized to produce controlled architectural and composition parts at the meso- and macro-scales for various applications. N. Nawafleh *et al.*^114^ claim that the fiber-to-polymer matrix adhesion in thermoset nanocomposites is much higher because the fibers are coated with a small quantity of surfactant, which functions as a chemical bridge to bind the fiber to the thermoset matrix. The liquid resin in thermoset nanocomposites has the ability to moisten the fiber surface and accelerate the chemical bonding process. For the additive manufacture of liquid thermoset materials, direct write technology takes the place of the polymer melting and solidification-based FFF process (Fig. 3c). When viscous pastes are strong enough to maintain their shape, rheology-modifying nano-particles, and fiber reinforcements are combined with liquid polymer resins to provide the right geometries. Ultimately, heat or light hardening the extruded material makes it tougher. Many research groups have embraced direct-write additive manufacturing since Compton^115^ and Lewis^116^ used it to create carbon-fiber-reinforced epoxy nanocomposites in 2014. This has allowed them to create a wide range of thermoset matrices (epoxy, cyanate ester, bismaleimide) reinforced with Kevlar or short carbon fibers.

R. Tandel^117^ and her colleagues investigated graphene flakes, polyethylene oxide nanocomposites, and microscale spherical Eutectic Gallium Indium (EGaIn) fillers that were used as solvent-based substrates (inks) for direct-ink-writing (DIW). The primary subjects of the findings being discussed are the effects of EGaIn fillers on the ink's rheology, the DIW process's operation, and the printed structures' electrical conductivity. The findings demonstrate how the rheology of the ink is changed by EGaIn fillers, giving it a more elastic behavior and a reduced extensional viscosity. This results in ink filaments that can print continuously even when printing speed surpasses ink flow speed, produce features with line widths less than nozzle diameter and withstand significant extensional strains during DIW. The strain increases lead the liquid EGaIn fillers along the printing path to deform, which lowers the prints' electrical conductivity. These results have the potential to control both the DIW procedure and the characteristics of the conductive polymer nanocomposites. EGaIn fillers in particular might be printed more quickly, at lower flow rates, and longer standoff distances, improving the dependability and efficiency of additive manufacturing in PC processing.

### 3.4. Chemical vapor deposition (CVD)

Chemical Vapor Deposition (CVD) relies on gas-phase chemical reactions to deposit metal layers onto polymer substrates. These reactions involve the decomposition of metal–organic precursors, leading to the formation of a thin metal film. Control over the chemical composition and reaction conditions is essential for achieving the desired film properties. Physical Vapor Deposition (PVD) involves the vaporization of metal targets in a vacuum environment, followed by condensation onto a polymer substrate. The chemical interactions between the metal atoms and the polymer surface play a crucial role in determining the adhesion, uniformity, and overall quality of the deposited film. For example, in PVD of aluminum (Al) onto a polymer, the vaporized Al atoms interact with the polymer surface, leading to a strong adhesive bond formed by van der Waals forces and potential chemical bonds,^118^ such as:Al + polymer surface → Al–polymer bond

CVD can be used to deposit thin films onto a substrate of materials such as silicon dioxide or silicon nitride, which can subsequently be patterned using photolithography or other processes. The intended patterns are then transferred onto the substrate using techniques like etching or deposition using these patterned masks. CVD is used in additive manufacturing, especially in the field of 3D printing, for a number of reasons, such as:

(i) Material deposition: Chemical Vapor Deposition 3D Printing (CVD-3DP) is one additive manufacturing technology that uses CVD techniques to deposit tiny layers of material onto a substrate or layers that have already been deposited. This makes it possible to precisely manipulate the composition and properties of the materials to create complex structures.^119^

(ii) Surface modification: the surface characteristics of 3D-printed items can also be changed using CVD. Coatings can be placed *via* CVD, for instance, to increase electrical conductivity, improve wear resistance, or offer other desired surface properties.^110^

(iii) Creation of support structures: in order to prevent deformation or collapse during printing, several additive manufacturing procedures call for the construction of support structures next to the main object. Sacrificial support materials can be deposited *via* CVD and then removed to reveal the desired object.^120^

(iv) Functionalization: by applying thin layers of functional materials to the surfaces of 3D-printed items, CVD can also be used to functionalize those products. Coatings for biocompatibility, resistance to corrosion, and other specific uses can fall under this category.^121^

**Fig. 4:**
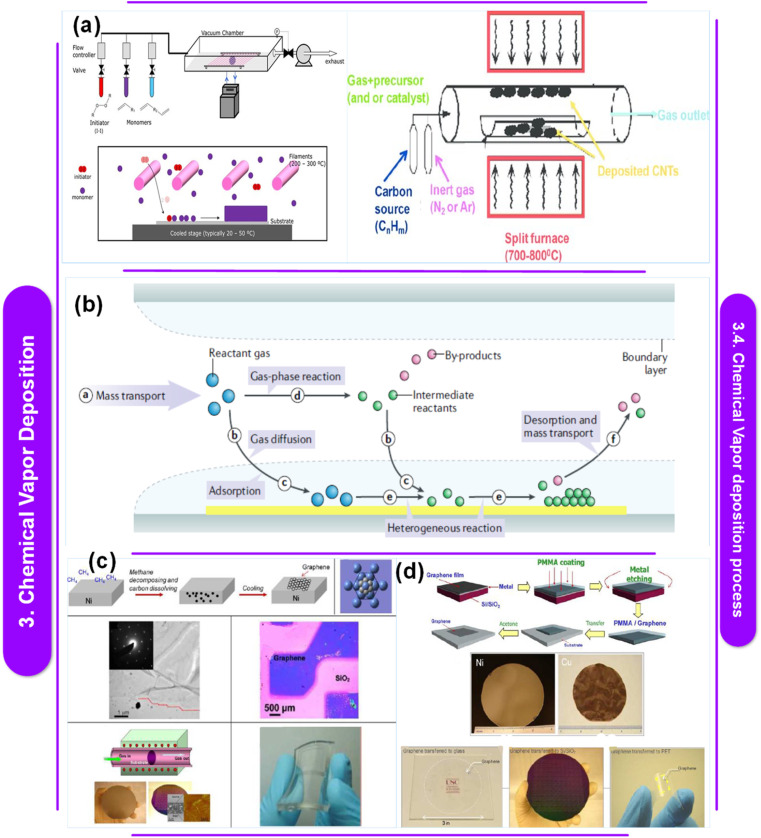
(a) Schematic illustration of the setup for chemical vapor deposition.^129^ This figure has been reproduced from ref. 129 with permission from InTech, copyright 2016. (b) Diagram showing the fundamental, generic processes in a typical CVD procedure.^130^ This figure has been reproduced from ref. 130 with permission from Springer Nature, copyright 2023. (c) Schematic representation showing the bigger atoms on the Ni (111) lattice and the smaller atoms of graphene. Low magnification TEM picture showing the edges of graphene. Optical picture of graphene across a SiO_2_/Si substrate from the Ni surface (d).^131^ This figure has been reproduced from ref. 131 with permission from the American Chemical Society, copyright 2013. Graphene layer deposition at full wafer scale on polycrystalline Ni films of flexible and transparent graphene on PDMS substrates. A flowchart representing the transfer procedure. Graphene synthesis on wafers using Cu foil and evaporated Ni film. Graphene films were deposited on top of a glass wafer, PET film, and device-designed Si/SiO_2_.

L. Sun *et al.*^122^ suggest that chemical vapor deposition (CVD) is the most effective method for producing solid thin films and coatings of superior quality. While widely utilized across various industries, CVD continues to evolve to accommodate the requirements of new materials. Today, CVD synthesis enables the precise synthesis of high-purity polymeric thin films applicable to diverse substrates, as well as inorganic thin films of 2D materials, pushing its capabilities to new heights. The primer includes an overview of the CVD technique, covering material characterization, process control, instrument building, and repeatability issues, as depicted in Fig. 4a and b.

Table 5 offers insights into different studies investigating the use of Chemical Vapor Deposition (CVD) techniques for synthesizing polymer–metal nanocomposites and related materials. Each entry provides a brief summary of the research paper's discoveries, detailing the CVD methods used, the materials involved, and any identified research gaps.^124,125^ These gaps range from the limited evaluation of surface treatments in polymer metallization using CVD to the need for reliable vapor deposition techniques for fabricating diverse conjugated polymer films. Overall, the table highlights ongoing efforts to advance CVD technologies for the production of functional polymer–metal nanocomposites while identifying areas for further investigation and development.

**Table 5:** Exploring advances and research gaps in chemical vapor deposition techniques for polymer–metal nanocomposites

Article title	Summary	Polymer–metal nanocomposite used by CVD	Research gaps	Challenges	Potential solutions	Ref.
Metallization of polymer nanocomposites by metalorganic chemical vapor deposition of Cu: surface functionalization driven film characteristics	Improving surface reactivity causes the deposit's center of mass to shift in the direction of the hot-wall reactor's gas entry	Polymer nanocomposite treated with various surface treatments before the deposition of Cu films using MOCVD	Limited MOCVD evaluation, insufficient polydopamine durability studies, need for surface reactivity research, and model integration for full process understanding	Limited MOCVD surface treatment evaluation, insufficient high-temp durability studies on polydopamine, need for research on surface reactivity in hot-wall reactors, and model integration for complete understanding	Conduct comprehensive studies on various surface treatments, explore the durability of polydopamine films at high temperatures, and develop models that incorporate surface reactivity to improve understanding and control of the deposition process in hot-wall reactors	123
Vapor phase organic chemistry to deposit conjugated polymer films on arbitrary substrates	Vapor deposition methods enable the film formation process to be significantly independent of substrate characteristics	Poly(thieno[3,2-*b*]thiophene)	Lack of reliable vapor deposition techniques for fabricating structurally diverse conjugated polymer films	Lack of reliable vapor deposition techniques for fabricating structurally diverse conjugated polymer films	Develop and refine vapor deposition techniques to reliably fabricate structurally diverse conjugated polymer films on various substrates	126
Nanocomposite metal amorphous-carbon thin films deposited by hybrid PVD and PECVD technique	To deposit nanocomposite DLC (diamond-like carbon) films, a hybrid method combining PVD (physical vapor deposition) employing magnetron sputtering and plasma enhanced chemical vapor deposition using CH_4_ gas was used	The research project produced nanocomposite coatings by embedding metals and ceramics in an amorphous carbon matrix, utilizing a hybrid PVD-PECVD technique for deposition	Approaches to mitigate stress, improve adhesion and compatibility with substrates, and attain a low friction coefficient and superior wear resistance in nanocarbon coatings	Approaches to mitigate stress, improve adhesion and compatibility with substrates, and attain a low friction coefficient and superior wear resistance in nanocarbon coatings	Investigate advanced hybrid PVD-PECVD techniques, and optimize parameters to reduce stress, enhance adhesion, and improve the overall performance of nanocarbon coatings	127

Electrical conductivity, which can be imparted to polymer nanocomposites by applying a metallic coating to their surfaces, is often required. Since wet chemicals are often environmentally hazardous, metalorganic chemical vapor deposition (MOCVD) offers a good alternative for treating complex, non-line-of-sight surfaces. The most common metal for this purpose is copper, which is the second least electrically resistant metal after silver.^128^ It also has a moderate environmental impact, is widely available, and is easily accessible. However, several restrictions may make the deposition process difficult because polymers in general and epoxies in particular have very low surface energies (between 20 and 50 mJ m^−2^, as opposed to 1000 mJ m^−2^ for metals and oxides).^132^ These include a significant delay in Cu nucleation, uneven and discontinuous film formation, and inadequate substrate adherence. How these shortcomings are fixed depends on two factors: the surface characteristics of the polymer substrate and the chemistry of the deposition process. Y. Zhang,^133^ a researcher who specializes in CVD and its applications, claims that the formation of monolayer and few-layer graphene is facilitated by the grain boundaries seen in polycrystalline Ni films. Zhang *et al.* employed single-crystalline Ni (111) substrates to improve the yield of monolayer graphene because they provide a grain-free, smooth surface (Fig. 4c). Because of the low solubility of carbon in Cu, Cu has emerged as a superior catalyst for the production of monolayer graphene with a substantial fraction of single layers. Whereas carbon segregation and precipitation can lead to the production of several layers on Ni surfaces, surface interactions on Cu surfaces drive the formation of monolayer graphene. The electrical properties of graphene created by CVD provide a wide range of potential uses. As shown in Fig. 4d, few-layer graphene grown on Ni, for instance, can be used to create transparent, flexible, and conductive electrodes for organic solar cells.

### 3.5. Physical vapor deposition (PVD)

**Fig. 5:**
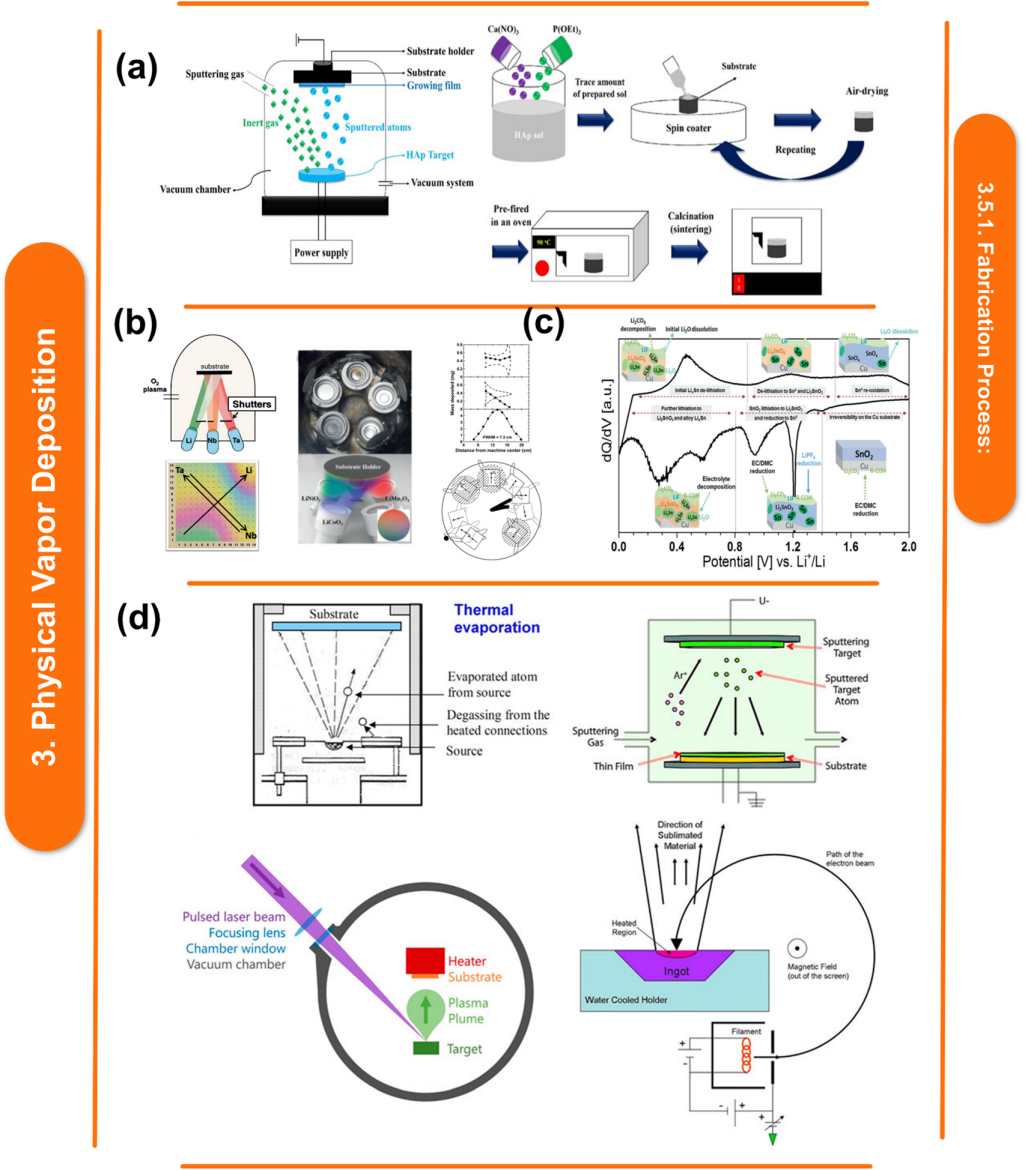
(a) Schematic representation of the HAp coating production process using spin coating.^141^ This figure has been reproduced from ref. 141 with permission from MDPI, copyright 2021. (b) Various technical configurations for PVD's creation of material libraries, configuration for interlayer deposition utilizing oxygen plasma and Knudsen-effusion cells (top), and the sample produced from Li–Ta–Nb (bottom).^142^ This figure has been reproduced from ref. 142 with permission from Wiley-VCH Verlag GmbH & Co. KGaA, copyright 2021. (c) Phase formation and reactions that take place at various potentials in relation to Li/Li^+^ during the lithiation and delithiation of a thin-film SnO_2_ electrode,^143^ This figure has been reproduced from ref. 143 with permission from American Chemical Society, copyright 2018. (d) Various techniques of physical vapor deposition are employed, including thermal evaporation, Ar gas sputter deposition, pulsed laser deposition, and electron beam evaporation.^144^ This figure has been reproduced from ref. 144 with permission from Frontiers Media SA, copyright 2021.

Physical Vapor Deposition (PVD) involves the vaporization of metal targets in a vacuum environment, followed by condensation onto a polymer substrate. The chemical interactions between the metal atoms and the polymer surface play a crucial role in determining the adhesion, uniformity, and overall quality of the deposited film.^118^ Metal atoms are evaporated from a solid source to create a vapor phase during PVD. A thin metal coating is subsequently formed on the polymer substrate by the condensation of these evaporated atoms. Metals are deposited onto polymer substrates using a variety of PVD techniques, including sputtering, evaporation, and ion plating. Each technique has its own advantages with regard to adhesion, uniformity, and deposition rate. PVD is a general term used to describe a variety of deposition techniques applied to electrically conductive surfaces to form thin films and protective coatings. A target, or condensed-phase material, is sputter- or evaporation-transformed into a vapor phase in a high vacuum chamber during the process. The resulting vapor phase is subsequently transported through an inert atmosphere to the atomic level. At some point, a condensed layer covers the substrate, as seen in Fig. 5a. PVD offers several advantages in metal–polymer nanocomposites, including fine-grained control over the film's composition, thickness, and homogeneity. Furthermore, by using this method, metals with specific characteristics can be deposited onto polymer substrates to improve their mechanical, electrical, and optical qualities.^20^

According to B. Uzakbaiuly *et al.*^134^ a number of techniques have been investigated for the successful co-integration of microdevices with microenergy sources as smart electronics have advanced. Physical vapor deposition, or PVD, (Fig. 5b) is one of the most promising techniques for this kind of work. It can be accomplished by a variety of techniques, such as thermal evaporation, ion-beam deposition, magnetron sputtering, pulsed laser deposition, *etc.* All solid-state thin-film batteries have undergone layer-by-layer deposition using solar deposition, resulting in a tremendous deal of work over the past 20 years. As these batteries have homogeneous active components and are usually binder-free, they have a higher potential energy density than batteries created using the traditional powder slurry approach (Fig. 5c). Magnetron sputtering is a frequently used method by ASTBs to deposit different materials for the cathode and anode. Reactive magnetron sputtering is mostly used for electrolyte deposition. With a chemical composition of Li_*x*_PO_*y*_N_*z*_, the lithium phosphorus oxynitride (Lipon) electrolyte was created at Oak Ridge National Laboratory (ORNL) and is known for being an extremely efficient electrolyte for lithium-ion batteries.^135^Fig. 5d highlights its exceptional stability in the air and steady performance up to 5 V *vs.* Li/Li^+^.

PVD-produced metal–polymer nanocomposites find extensive use in industries such as biomedicine, electronics, aerospace, and renewable energy. These nanocomposites have unique properties that make them suitable for a variety of applications, including coatings, sensors, electronic devices, and medical implants.

### 3.6. State-of-the-art fabrication techniques

In the realm of polymer–metal nanocomposites, particularly for energy applications, there has been considerable emphasis on established fabrication techniques such as Chemical Vapor Deposition (CVD) and Physical Vapor Deposition (PVD). While these methods are well-documented and widely used, emerging techniques like electrohydrodynamic (EHD) processing and aerosol jet printing present innovative alternatives that could offer significant advantages in this field.

#### 3.6.1. Electrohydrodynamic (EHD) processing

Electrohydrodynamic (EHD) processing is a technique that utilizes electric fields to manipulate and deposit fluids containing nanomaterials onto substrates. One of the primary benefits of EHD processing is its ability to produce highly uniform and finely controlled patterns, which is particularly valuable for applications that demand precision, such as in energy storage devices. The technique can handle a variety of materials, including polymers and metals, and operates at relatively low temperatures, preserving the integrity of sensitive materials. However, EHD processing is not without its challenges. The process can be relatively slow, particularly for large-scale applications, which can limit its industrial scalability. Additionally, it is sensitive to environmental conditions like humidity and temperature, which can affect the consistency of the deposited patterns. There is also a risk of nozzle clogging, which can disrupt the process and lead to defects in the final product.^152^

Despite these challenges, EHD processing holds significant promise for energy applications. For instance, in the fabrication of batteries and supercapacitors, the ability to create intricate electrode structures can enhance surface area and improve charge storage capacity. To overcome the limitations of EHD processing, researchers are exploring the use of parallelized systems with multiple nozzles to increase throughput, as well as controlled environments to ensure consistent deposition quality.

#### 3.6.2. Aerosol jet printing

Aerosol jet printing is another emerging technique that offers unique advantages for the fabrication of polymer–metal nanocomposites. This additive manufacturing method allows for the direct writing of functional materials onto a variety of substrates, including flexible and curved surfaces, making it particularly suitable for the development of flexible energy devices. Aerosol jet printing can handle a wide range of materials, including metal nanoparticles and composite inks, enabling the creation of multi-material structures in a single fabrication process. This versatility is key for applications that require precise placement of materials, such as in thin-film transistors and other microelectronic components. However, aerosol jet printing also faces certain limitations. The stability of the inks used in this process can be a concern, as particles within the ink can agglomerate or settle, leading to inconsistent deposition and defects in the printed features. The resolution of aerosol jet printing, while generally good, may not be sufficient for certain high-precision applications. Additionally, similar to EHD processing, the speed of aerosol jet printing can be a limiting factor, particularly for large-area or complex patterns.^153^

To address these challenges, advancements in ink formulation, such as the use of stabilizers to prevent particle agglomeration, are being explored. Continuous agitation of the ink during printing can also help maintain uniformity. Improvements in nozzle design and the development of finer aerosol streams are being investigated to enhance resolution. Furthermore, increasing the speed of the printing process is a key area of focus, with multi-nozzle systems being one potential solution. In summary, while both EHD processing and aerosol jet printing have their respective limitations, they also offer significant opportunities for advancing the fabrication of polymer–metal nanocomposites in energy applications. By addressing the current challenges associated with these techniques, it is possible to develop innovative energy devices that offer enhanced performance and new functionalities, contributing to the ongoing evolution of sustainable energy technologies.

#### 3.6.3. Nanolithography

Nanolithography is a technique used to create extremely fine patterns at the nanoscale, which is particularly critical in the development of polymer–metal nanocomposites for electronic applications. Traditional lithography techniques, while effective for larger-scale patterns, fall short when precision at the nanometer scale is required. Nanolithography enables the precise placement of metal nanoparticles within a polymer matrix, thereby allowing for the design of nanocomposites with tailored electrical and mechanical properties. This technique uses a variety of approaches, including electron-beam lithography (EBL), nanoimprint lithography (NIL), and scanning probe lithography (SPL). EBL, for instance, employs a focused beam of electrons to directly write patterns on a resist material, which is then developed to reveal the desired nanoscale features. NIL involves pressing a hard mold with nanoscale features into a soft polymer layer, transferring the pattern with high fidelity. These methods allow for the fabrication of complex structures such as nanowires and nanoarrays, which are essential for applications in nanoelectronics, sensors, and flexible electronics. Nanolithography's ability to control the placement and distribution of metal nanoparticles within the polymer matrix enhances the electrical conductivity and mechanical strength of the resulting nanocomposite films. This precision is especially important in applications where the interaction between the metal and polymer phases needs to be finely tuned to achieve optimal performance.^154^

#### 3.6.4. Advanced laser-assisted deposition

Advanced laser-assisted deposition techniques, such as Pulsed Laser Deposition (PLD) and Laser-Induced Forward Transfer (LIFT), have evolved to become pivotal in the fabrication of high-quality polymer–metal nanocomposite films. These methods use laser energy to vaporize materials and deposit them onto a substrate, offering unparalleled control over film thickness, uniformity, and adhesion. In PLD, a high-power laser pulse is directed at a target material, causing it to ablate and form a plasma plume. The material from the plume then deposits onto a substrate, forming a thin film. This technique is particularly advantageous for depositing multi-component materials, as it preserves the stoichiometry of the target material in the deposited film. LIFT, on the other hand, uses laser pulses to transfer material from a donor film to a substrate in a precise and controlled manner, making it ideal for patterning complex nanocomposite films without the need for masks or additional processing steps. Advanced laser-assisted deposition is highly versatile, allowing for the fabrication of films with tailored optical, electrical, and mechanical properties. It is especially useful in applications requiring high precision, such as in the production of flexible electronics, sensors, and photovoltaic devices. The ability to control the deposition environment (*e.g.*, temperature, atmosphere) further enhances the quality and performance of the nanocomposite films produced.^155^

#### 3.6.5. Atomic layer deposition (ALD)

Atomic Layer Deposition (ALD) is a vapor-phase technique that deposits thin films one atomic layer at a time. This method offers precise control over film thickness and composition, making it ideal for fabricating polymer–metal nanocomposites with highly uniform coatings. ALD is particularly beneficial for coating substrates with complex geometries, as it ensures conformal coverage over all surfaces, including deep trenches and high-aspect-ratio structures. In the ALD process, a substrate is exposed to alternating pulses of precursor gases, which react with the surface in a self-limiting manner. This process ensures that only a single atomic layer is deposited during each cycle, allowing for precise control over the film's thickness at the angstrom level. By carefully selecting the precursor materials and reaction conditions, ALD can be used to deposit a wide range of materials, including metals, oxides, and nitrides, onto polymer substrates. ALD is particularly valuable in applications where uniformity and control at the atomic scale are crucial, such as in the fabrication of high-performance capacitors, transistors, and barrier coatings. The method's ability to deposit ultrathin films with exceptional uniformity also makes it ideal for creating polymer–metal nanocomposites used in flexible electronics and advanced energy storage devices.^156^

#### 3.6.6. Electrohydrodynamic (EHD) jet printing

Electrohydrodynamic (EHD) jet printing is an advanced printing technique that utilizes electric fields to produce ultra-fine jets of material, which can be precisely deposited onto a substrate. Unlike traditional inkjet printing, which relies on the mechanical ejection of droplets, EHD jet printing uses an electric field to pull the liquid out of the nozzle, allowing for the creation of much finer droplets and thus higher resolution patterns. This technique is particularly effective for depositing materials at the micro- and nanoscale, making it suitable for fabricating intricate polymer–metal nanocomposite films. EHD jet printing allows for the deposition of functional materials, including metals, polymers, and nanoparticles, onto a variety of substrates. The process can be tuned to control the size of the droplets and the thickness of the deposited layers, providing precise control over the final nanocomposite structure.^157^

EHD jet printing is ideal for applications that require high precision and fine patterning, such as in the production of microelectronics, sensors, and wearable devices. The ability to print complex structures with high resolution makes it a powerful tool for creating next-generation polymer–metal nanocomposites with enhanced functionality.

#### 3.6.7. Roll-to-roll processing

Roll-to-Roll (R2R) processing is a high-throughput fabrication technique that enables the continuous production of polymer–metal nanocomposite films on flexible substrates. This method involves unwinding a roll of substrate material, passing it through various processing stations, and then rewinding it onto another roll. The process can include multiple steps, such as coating, drying, patterning, and laminating, all of which are performed in a continuous and automated manner. R2R processing is particularly advantageous for large-scale production, as it allows for the rapid fabrication of nanocomposite films with consistent quality. The technique is highly scalable and cost-effective, making it ideal for applications that require large-area films, such as in flexible electronics, solar cells, and wearable devices. Additionally, R2R processing can be integrated with other advanced techniques, such as ALD or EHD jet printing, to enhance the functionality and performance of the final product. One of the key benefits of R2R processing is its ability to produce multilayered nanocomposite films with precisely controlled thickness and composition. This capability is critical for developing advanced polymer–metal nanocomposites with tailored electrical, optical, and mechanical properties.^158^

#### 3.6.8. Integration of machine learning in fabrication

The integration of Machine Learning (ML) in fabrication processes represents a significant leap forward in the development of polymer–metal nanocomposites. By incorporating ML algorithms, manufacturers can optimize fabrication parameters in real-time, leading to improved efficiency, consistency, and performance of the final product. ML can be used to analyze large datasets generated during the fabrication process, identifying patterns and correlations that may not be immediately apparent through traditional analysis methods. For example, ML algorithms can optimize the deposition rate, temperature, and chemical composition during techniques such as ALD or EHD jet printing, ensuring that the final nanocomposite films meet precise specifications. Furthermore, ML-driven fabrication processes can adapt to variations in the input materials or environmental conditions, automatically adjusting parameters to maintain consistent quality. This level of control is particularly valuable in high-precision applications, such as in the development of flexible electronics, sensors, and energy storage devices. The integration of ML in fabrication not only enhances the quality and performance of polymer–metal nanocomposites but also reduces waste and production costs. As the field of ML continues to advance, its application in fabrication processes is expected to become increasingly sophisticated, leading to even greater innovations in nanocomposite materials.^159^

Table 6 provides a comparative overview of advanced fabrication techniques for polymer–metal nanocomposites, focusing on their applications in energy-related fields. Techniques such as electrohydrodynamic (EHD) processing and aerosol jet printing are highlighted for their precision and versatility, though they face challenges like scalability, environmental sensitivity, and ink stability. Nanolithography offers nanoscale precision but is limited by its complexity and cost, while advanced laser-assisted deposition provides control over film properties but is hindered by high energy consumption. Atomic Layer Deposition (ALD) excels in uniformity and thin-film control, yet it struggles with slow deposition rates. EHD jet printing is effective for micro and nanoscale applications but shares some of the limitations of EHD processing. Roll-to-Roll (R2R) processing is noted for its scalability and cost-effectiveness in large-area film production, though it requires precise control. The integration of machine learning in fabrication processes is seen as a promising approach to enhance efficiency and precision, despite the need for extensive data and computational resources. Each technique's strengths, limitations, and potential solutions are discussed, with references provided for further exploration.

**Table 6:** Overview of advanced fabrication techniques for polymer–metal nanocomposites in energy applications

Technique	Pros	Cons	Applications	Limitations	Potential solutions	Ref.
Electrohydrodynamic (EHD) processing	High precision, uniform patterns	Slow process, limited scalability	Energy storage devices (*e.g.*, batteries, supercapacitors)	Sensitive to environmental conditions (humidity, temperature), nozzle clogging	Parallelized systems, controlled environments, anti-clogging mechanisms	152
	Low-temperature operation, versatile material handling		Creating intricate electrode structures			
Aerosol jet printing	Direct writing on flexible/curved surfaces	Ink instability, potential for defects	Flexible electronics, microelectronics, thin-film transistors	Resolution may be insufficient for some high-precision applications, slow for large areas/patterns	Advanced ink formulations, continuous ink agitation, improved nozzle design, multi-nozzle systems	153
	Handles a wide range of materials, multi-material structures in one process		Precise placement of conductive materials			
Nanolithography	Nanoscale precision, tailored electrical/mechanical properties	Complex and expensive equipment, time-consuming	Nanoelectronics, sensors, flexible electronics	Limited scalability for large-scale production	Combining with scalable techniques like roll-to-roll processing, advancements in lithography technology	154
	Enables the creation of nanowires, nanoarrays					
Advanced laser-assisted deposition	High control over film thickness, uniformity, and adhesion	High energy consumption, potential material wastage	Flexible electronics, sensors, photovoltaic devices	Equipment costs and operational complexity, limited scalability	Integration with R2R processing, optimization of laser parameters to reduce energy consumption	155
	Ideal for multi-component materials, no need for masks		High-precision thin films			
Atomic layer deposition (ALD)	Precise control over film thickness, uniform atomic layers	Slow deposition rates, high equipment costs	High-performance capacitors, transistors, barrier coatings	Limited to thin films, complex geometries can be challenging	Developing faster ALD processes, optimizing precursor materials for quicker deposition	156
	Conformal coverage on complex geometries		Flexible electronics, advanced energy storage			
Electrohydrodynamic (EHD) jet printing	Ultra-fine jet printing, high-resolution patterning	Nozzle clogging, environmental sensitivity	Microelectronics, sensors, wearable devices	Requires highly controlled environments for consistency	Anti-clogging measures, controlled environments, optimized fluid formulations	157
	Suitable for micro/nanoscale deposition		Intricate polymer–metal nanocomposite structures			
Roll-to-roll (R2R) processing	High-throughput, scalable, cost-effective	Limited to thin, flexible substrates, challenges with multi-layer alignment	Flexible electronics, solar cells, wearable devices	Precision control can be difficult, alignment of multi-layered structures	Integration with other advanced techniques (*e.g.*, ALD, EHD), improved alignment technologies	158
	Continuous production with consistent quality		Large-area film production			
Integration of machine learning in fabrication	Real-time optimization of fabrication parameters, improved efficiency	Requires large datasets, high computational resources	Flexible electronics, sensors, energy storage devices	Initial setup and data collection can be time-consuming, high complexity	Developing ML models tailored to specific fabrication processes, real-time data analysis capabilities	159
	Adaptive to variations, reduces waste and costs		Precision fabrication processes			

## 4. Polymer–metal nanocomposites for energy applications

Polymer–metal nanocomposite energy harvesting has become a viable approach in the search for economical and sustainable energy sources.

### 4.1. Role of polymer metal nanocomposites for energy harvesting and storage

Energy harvesting is the technique of gathering and storing minute amounts of energy for later use from one or more nearby energy sources. Other names for energy harvesting include power harvesting and energy scavenging. Extracting and converting ambient energy into useable electrical power is the aim of energy harvesting technologies. Because of their special combination of dynamic flexibility, electrical resistance, and customizable properties, polymer–metal nanocomposites have attracted a lot of attention.^139^

#### 4.1.1. Vibration energy harvesting

**Fig. 6:**
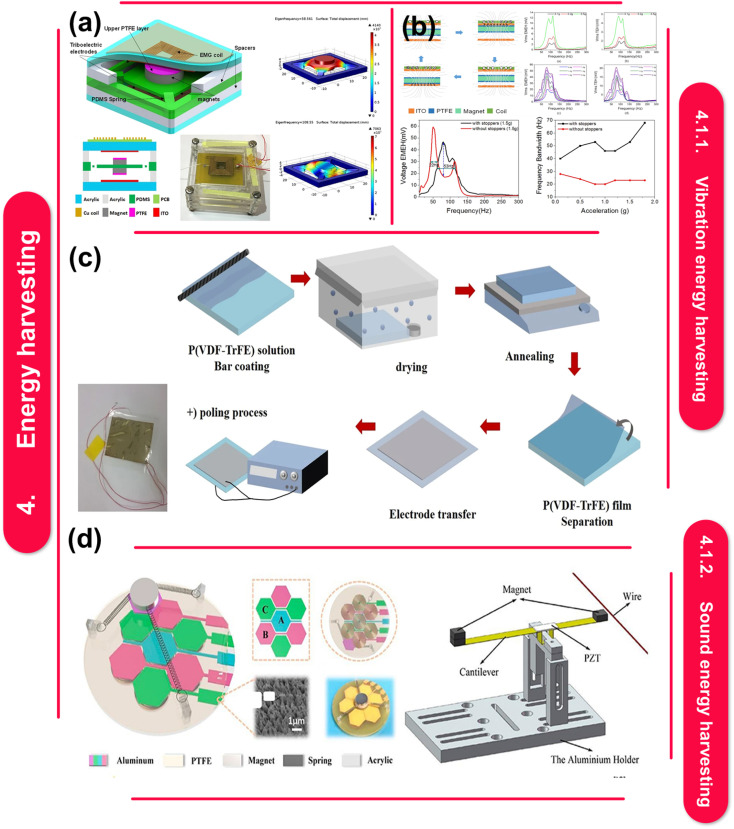
(a) Broadband and hybrid energy harvester (B-HEH) schematic drawing; reproduced with permissions. Image of the manufactured device prototype; FEM simulation performed with COMSOL.^136^ This figure has been reproduced from ref. 136 with permission from Nature Publishing Group, copyright 2017. (b) Procedures for producing voltage by electromagnetic and triboelectric mechanisms; frequency spectra showing multimodal behavior for the electromagnetic and triboelectric output voltages at 0.1, 0.2, and 0.5 g.^137^ This figure has been reproduced from ref. 137 with permission from Nature Publishing Group, copyright 2017. (c) Single-sided graphene transfer-based G/PVDF/G generators and P(VDF-TrFE) based thin film generators (a picture of a manufactured device with silver electrodes is included in the inset).^140^ This figure has been reproduced from ref. 140 with permission from Nature Publishing Group, copyright 2022. (d) Diagram of a three-electrode, triboelectric–electromagnetic hybrid energy harvester for ocean waves, shaped like a honeycomb.^140^ This figure has been reproduced from ref. 146 with permission from IOP Publishing, copyright 2020.

Utilizing the piezoelectric qualities of polymer–metal nanocomposites, vibration energy harvesting is possible. These nanocomposites use the piezoelectric effect to produce electrical charges in response to mechanical vibrations. This phenomenon makes it possible to convert mechanical energy from vibrations into electrical energy, which makes it a useful technique for sensors and tiny electronic devices. The precise selection of nanocomposite materials made of polymers and metals is essential to the effectiveness of vibration energy collection. When paired with metal elements like zinc oxide or aluminum, polymers with high piezoelectric coefficients, such as polyvinylidene fluoride (PVDF), improve energy conversion efficiency. The nanocomposite's total ability to capture and transform vibrational energy is determined by the synergy between the metal components and polymer matrix. Because piezoelectric materials can convert mechanical vibration into electrical energy with simple designs, piezoelectric energy harvesting is highlighted as an independent power source for wireless sensor network systems (Fig. 6a and b). When mechanical stress or vibrational effects cause piezoelectric materials to undergo dimensional changes, the materials act as sensors and produce an electrical voltage. When an alternate voltage is provided to piezoelectric materials, they vibrate at a frequency equivalent to the applied voltage, acting as actuators. Because of its self-powered qualities during manufacture, polymer-based nanofiber sensors utilizing piezoelectric properties fabricated through electrospinning have gained increasing prevalence, especially in oscillatory energy harvesting technologies. They also don't require additional electrical polarization. There is an investigation into oscillatory energy storage in the PEN involves testing under various resistive loads. When a resistive load of 1 MΩ is applied at a frequency of 15 Hz, the PEN featuring PVDF panels with 10 vol% PZT exhibits a maximum output power of 6.35 μW at 85% power. In contrast, the PEN with β-PVDF yields an electrical power of 3.44 μW under identical load conditions.^140^ Without requiring an external power source, this PEN-based energy generation system offers a workable way to produce clean energy from mechanical vibrations in mobile microelectronic applications (Fig. 6c).

#### 4.1.2. Sound energy harvesting

We refer to the process of converting acoustic vibrations, or sound waves, into electrical energy as “sound energy harvesting.” Polymer–metal nanocomposites can be designed to exhibit piezoelectric properties, meaning that sound waves can cause them to generate electrical charges. Sound-energy-harvesting membranes or thin films that vibrate in response to sound waves can be made using polymer–metal nanocomposites. When sound waves come in contact with the nanocomposite, mechanical deformation results in an electrical polarization, which generates an electric charge. Potential applications for polymer–metal nanocomposites for sound energy harvesting include environmental sensing, wireless communication, smart buildings, and noise pollution monitoring. Through the use of polymer–metal nanocomposites, ambient sound energy may be converted into electrical power, opening up new environmentally friendly and self-sufficient energy sources for a variety of applications. An acoustic energy harvesting system is typically made up of an acoustic resonating component and an energy conversion component that uses electromagnetic or piezoelectric materials. Two types of studies are usually conducted when it comes to using acoustic resonators for energy harvesting: (1) using traditional resonators like the quarter-wave resonator and Helmholtz resonator, and (2) using artificial acoustic structures like photonic crystals and metamaterials.^145^

According to M. Yuan *et al.*^146^ acoustic energy refers to a form of energy present in the environment that can be harnessed and transformed into electrical energy for utilization in small-scale power initiatives. Acoustic energy harvesting (AEH) in structural design requires meticulous attention to detail due to the sometimes-low levels of incoming sound power density. AEH equipment is necessary to transform acoustic energy into electrical energy efficiently (Fig. 6d). This device has the capability to utilize electromagnetic, piezoelectric, or triboelectric phenomena in order to concentrate incoming acoustic energy and convert it into useable energy. A power management circuit carries out voltage rectification, control, and impedance matching following conversion.

In conclusion, the exploration of sound energy harvesting represents a significant advancement in the field of renewable energy generation. By leveraging polymer–metal nanocomposites with piezoelectric properties, sound waves can be effectively converted into electrical energy. This opens up a plethora of potential applications, ranging from environmental sensing to wireless communication and noise pollution monitoring. The integration of acoustic resonators and energy conversion components further enhances the efficiency of energy harvesting systems, allowing for the effective utilization of ambient sound energy. With careful attention to design and the incorporation of power management circuits, these systems hold promise for powering small-scale initiatives in a sustainable and environmentally friendly manner. As research in this area continues to evolve, the practical implementation of sound energy harvesting technologies is poised to revolutionize various sectors and contribute to a greener future.

### 4.2. Photovoltaic devices

Photovoltaic devices, often known as solar cells, are gadgets that use sunshine to generate electricity directly.^138^ Polymer–metal nanocomposites have garnered significant interest in the field of photovoltaics due to their potential for lightweight, low-cost, and flexible solar cell technologies.^147^

#### 4.2.1. Polymer solar cells

**Fig. 7:**
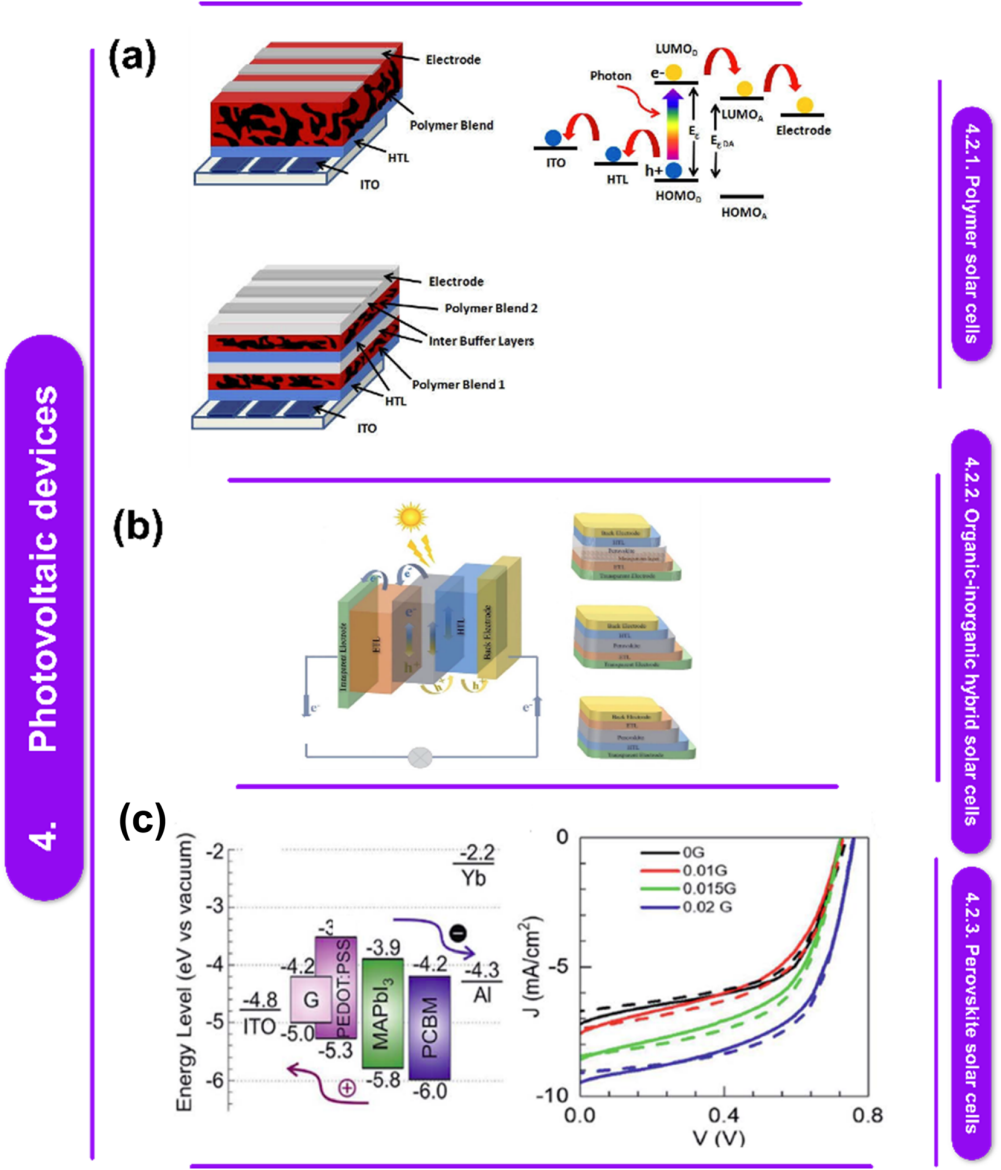
(a) The diagram illustrates the energy levels of a single junction BHJ cell, tandem junction BHJ architecture, and single junction BHJ architecture.^148^ This figure has been reproduced from ref. 148 with permission from Frontiers Media SA, copyright 2021. (b) Methods for preparing carbon nanomaterial polymer nanocomposites include solution mixing and *in situ* polymerization.^149^ This figure has been reproduced from ref. 149 with permission from The Royal Society of Chemistry, copyright 2022. Reproduced with permissions. (c) Current–voltage performances under one sun illumination for solar cells based on different materials as HTLs are shown in forward and reverse sweeps.^150^ This figure has been reproduced from ref. 150 with permission from Springer Nature, copyright 2023.

A polymer solar cell with single junction BHJ architecture is shown in Fig. 7a. Gusain *et al.*^148^ show that a photovoltaic device can immediately transform an absorbed photon's energy into an electrical charge. The way this device works is that it uses a semiconductor component whose electronic gap is either the same as or less than the energy of the photon (*hν*) that is being absorbed, which causes an electron–hole pair to be created. The released carriers of charge are subsequently transferred to their corresponding electrodes, where an internal field created by the different work functions of the electrodes traps them. An extremely thin layer that absorbs light, the active layer in a bulk heterojunction solar cell (BHJ) usually consists of a molecule with a high electron affinity combined with a conjugated polymer to create a nanostructured mixture. The organic BHJ device shown in Fig. 7a has a single connection and is composed of multiple layers: an active layer that combines donor and acceptor molecules; an indium-tin oxide (ITO) transparent bottom electrode; a hole transport layer (HTL) such as PEDOT:PSS(poly(3,4-ethylene dioxythiophene): polystyrene sulfonate); and a top electrode layer that is typically metallic. On the other hand, numerous stacks of single junction bulk heterojunction (BHJ) cells, each with a different configuration of donor/acceptor active layers, make up a tandem junction BHJ cell. To balance the charge transfer between the two cells, interlayers (ILs) are used.

The donor polymer in the active layer is excited by incident light photons to its Lowest Unoccupied Molecular Orbital (LUMO), which results in the creation of an exciton, which is a pair of bound electrons and holes because of Coulomb interactions. As soon as the exciton reaches the donor/acceptor contact, it diffuses through the donor polymer until it dissociates, which is enabled by an energetically advantageous electron transfer from the LUMO of the donor polymer to the LUMO of the fullerene acceptor. Heat and vibrations are released as a result of the energy differential between the donor–acceptor LUMOs during this phase.^150^ Then, the hole in the Highest Occupied Molecular Orbital (HOMO) of the donor polymer is moved through the material, gathered at the Hole Transport Layer (HTL), and transferred over the HTL/ITO electrode interface. The electron passes through the acceptor phase and across the connection between the acceptor and the metal electrode in the interim. Reducing these energy offsets has a direct effect on the open-circuit voltage and, in turn, the power conversion efficiency of the solar cell. The favorable hole transfer from the acceptor's HOMO to the donor's HOMO, which occurs if the light is absorbed in the acceptor molecule, controls exciton separation and initiates the first phase of the cascade of charge transfer.^135^

#### 4.2.2. Organic–inorganic hybrid solar cells

An organic–inorganic hybrid solar cell combines organic elements like polymers or small organic molecules with inorganic elements like metal oxides or nano-particles. Combining organic and inorganic components aims to improve the performance and efficiency of solar energy conversion by maximizing the advantages of each material type. According to J. Niederhausen,^151^ a hybrid solar cell uses both organic and inorganic semi-conductors (OSCs/ISCs) to convert solar light into power through the photovoltaic effect. Naturally, interfaces that arise between the realms of inorganic and organic materials regulate many of the fundamental properties of these devices; these interfaces are called hybrid interfaces. Hybrid interfaces are often employed to improve particular interface functionalities or to facilitate actions that would not be feasible in an on-hybrid configuration (Fig. 7b).

Because of their almost infinite chemical structural flexibility, organic materials can have precisely controlled chemical, electrical, and mechanical properties. This makes it possible to combine selective charge transport, effective passivation, and controlled surface bonding in one molecule. Additionally, it allows for changes to the solubility properties, opening a wide range of processing options. In the long run, solar energy is expected to meet a significant portion of the global electricity demand. The sheer volume of solar cell manufacturing requires replacing solar cell components made of rare resources like gallium and indium with more earthly elements to produce the terawatts of electricity needed.^152^ Since carbon and hydrogen make up most organic components, substituting their inorganic counterparts in solar cells can help with some of the material scarcity problems. Moreover, the introduction of novel materials with lower processing temperatures or at lower costs has the potential to lessen the energy and cost requirements for recycling and manufacturing. Such developments can hasten the shift to a sustainable way of living and increase the economic and environmental benefits of solar cells.

#### 4.2.3. Perovskite solar cells

In the realm of photovoltaics, perovskite solar cells (PSCs) are a promising and quickly developing technology with the potential to completely transform the solar energy industry. The light-absorbing layer of these solar cells is made of perovskite materials, which are usually hybrid organic–inorganic lead halide structures. The incorporation of carbon nanotubes and polymers into perovskite solar cells has become a viable and significant way to improve the stability and performance of these cells in recent years. Effective PSCs demand a superior perovskite thin film with an immense grain size and a steady crystal orientation, low defect concentration, and high surface coverage.^153^Fig. 7c depicts the various PSC device topologies as well as the overall PSC operation concept. The combination of perovskite materials, carbon nanomaterials (such as carbon nanotubes or graphene), and polymers offers a synergistic remedy for some of the issues associated with traditional PSCs. Because of their superior chemical stability, mechanical strength, and electrical conductivity, carbon nanomaterials can be used as efficient charge transport and collecting layers to reduce the effects of charge recombination and increase overall device efficiency.^154^

Conversely, polymers help solar cells remain stable and flexible, which increases their resilience and versatility in a range of applications. Polymer incorporation can also promote better film formation throughout the fabrication process, which will enhance the uniformity and quality of the perovskite film. Perovskite materials, carbon nanomaterials, and polymers work together synergistically to address issues like moisture-induced perovskite deterioration, thermal instability, and overall device lifespan. With the use of this interdisciplinary approach, a more effective, reliable, and commercially viable solar cell technology can be produced by utilizing the special qualities of each component. As this field of study develops, engineers and scientists are investigating novel approaches for the mass-production of carbon nanomaterial–polymer nanocomposite-based perovskite solar cells. The incorporation of these cutting-edge materials not only improves the solar cells' basic performance but also creates opportunities for transparent and flexible solar systems, opening up a wide range of uses for energy harvesting and storage. The secret to opening up the next generation of solar technologies with increased stability, efficiency, and versatility lies in the complex interactions between these components.^153^

### 4.3. Thermoelectric generators

**Fig. 8:**
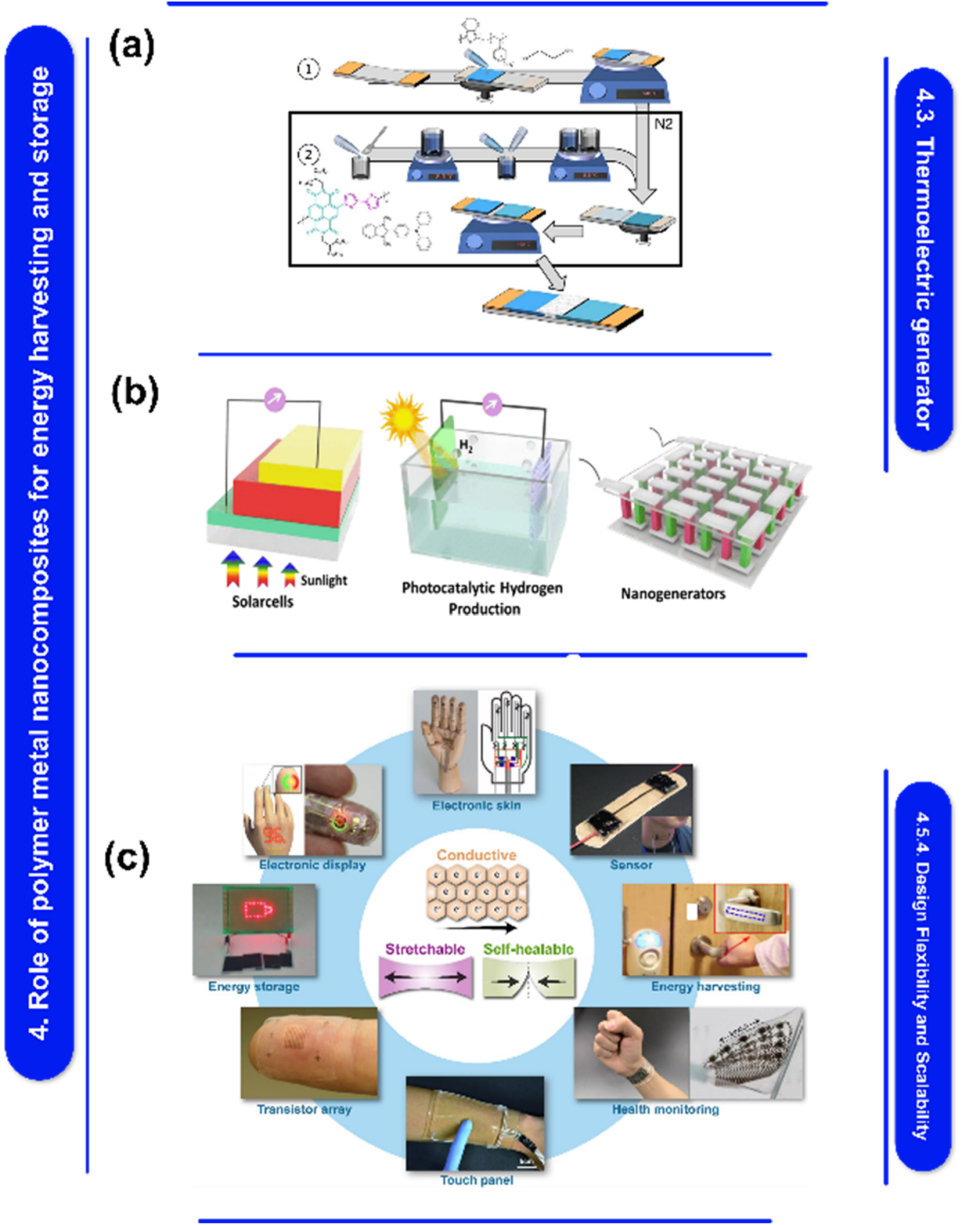
(a) Thin-film thermoelectric generator based on polymers. Diagram showing how an all-polymer TEG sample is prepared for solution processing, all-polymer TEGs' performance.^169^ This figure has been reproduced from ref. 169 with permission from Springer Nature, copyright 2023. (b) A schematic depiction of the different polymer nanocomposites and the energy uses for them is placed, a typical thermoelectric device's structure, which includes both single and multiple unicouple diagram illustrating the PVDFHP/NaTFSI nanocomposite's component distribution during thermoelectric production.^170^ This figure has been reproduced from ref. 170 with permission from MDPI, copyright 2021. (c) An example of stretchable and self-healing conductors and their possible uses, mechanisms of self-healing, examples of healable conductive electrodes, diagram showing the spread of a crack, the release of a healing agent, and the crack's repair.^171^ This figure has been reproduced from ref. 171 with permission from Wiley-VCH Verlag GmbH & Co. KGaA, copyright 2021.

Thermoelectric generators (TEGs) are devices that directly convert thermal energy into electrical power by leveraging the Seebeck effect. The Seebeck effect causes electrons to flow across the metal–polymer nanocomposite when a temperature gradient is provided, which produces an electric current. The basis for using waste heat as a possible source of sustainable energy is provided by this phenomenon. Metal–polymer nanocomposites have clear benefits for creating effective thermoelectric generators. Their capacity to merge the flexibility and lightweight qualities of polymer matrices with the high heat conductivity of metallic components produces a special combination of attributes (Fig. 8a). This combination is necessary to maximize heat transfer and electron flow efficiency within the nanocomposite, improving the thermoelectric generator's overall performance.^155^

Thermoelectric modules have the ability to convert thermal energy into electrical power, according to R. M. Kluge *et al.*^156^ By using inexpensive and readily available organic materials, they can use the Seebeck effect to produce energy from natural heat sources and low-grade waste heat in an environmentally responsible manner. Furthermore, organic materials' flexibility can enable them to be adapted to curved surfaces, like the skin of humans and wearable electronics. This article presents a solution-processable thermoelectric generator (TEG) that uses polymers alone for its active components. An n-type polymer with high mobility is [*N*,*N*-bis(2-octyldodecyl)-naphthalene-1,4,5,8-bis(dicarboximide)-2,6-diyl]. [2,2-iodopamine]-*alt*-5,5-. The well-studied p-type polymer blend poly(3,4-ethylenedioxythiophene): poly(styrenesulfonate) (PEDOT:PSS) and (P(ND_I2_OD-T_2_)) combine to form a thin-film TEG. The stated device design is not exclusive to this system; it can be used with any pair of organic components that can be separated from the solution and processed. As seen in Fig. 8a, it is also relatively simple to upgrade to fully flexible devices employing techniques like printing and roll-to-roll processing.

In summary, the employment of metal–polymer amalgamates within thermoelectric generators emerges as a promising avenue for harnessing waste heat and transforming it into electrical energy. The amalgamation of polymer matrices' flexibility and lightness with the superior heat conductivity of metallic constituents facilitates the development of efficient thermoelectric modules. Additionally, the introduction of solution-processable thermoelectric generators employing organic materials offers exciting prospects for sustainable energy generation. With ongoing advancements in materials science and manufacturing methods, such as printing and roll-to-roll processing, the realization of fully flexible and wearable thermoelectric devices becomes increasingly attainable. As research in this domain progresses, the practical application of thermoelectric generators across various sectors, from portable electronics to industrial contexts, promises to contribute to a greener and more sustainable future.

Thermoelectric materials enable the direct conversion of waste thermal energy into electrical energy, offering a solution to energy-related challenges. Polymer-based materials have been investigated for heat conversion in the temperature range of 20 to 200 °C, where conventional materials are less efficient. Polymers are thought to be potential materials due to their good electronic transport properties, non-toxicity, abundance, ease of processing, flexibility, and ease of adjustment. Metals can be introduced to polymer matrices to successfully minimize the negative impacts of their high heat conductivity on the resulting nanocomposites, thanks to their inherent high electrical conductivity. The TE modules from which TE devices or generators are built consist of P-type and/or n-type TE legs. The thermoelectric effect, especially the Seebeck effect, which turns heat into electricity, is the most effective means of using waste heat energy. When a material's two opposite sides encounter a temperature disparity, a potential difference is formed. Metals create a potential difference that allows charge carriers to move in a closed circuit or electric current. In evaluating thermoelectric (TE) materials, the dimensionless *ZT* = (*σ*·*S*^2^)/*k*·*T* is used, where *S* represents the Seebeck coefficient, *σ* denotes electrical conductivity, *k* represents thermal conductivity, and *T* is the absolute temperature. The performance of TE materials is occasionally assessed using just the numerator (PF, or power factor) of the previous equation in cases where *k* cannot be measured. It is only possible to reach maximal effectiveness when PF values are high and *k* values are low.^157^

### 4.4. Energy storage devices

The concepts of electrostatic double-layer capacitance and pseudo-capacitance underpin the operation of supercapacitors, also referred to as ultracapacitors. Because of their large surface area, superior electrical conductivity, and customized porosity all of which are critical for improving energy storage performance, metal–polymer nanocomposites are a promising alternative for electrode materials. In energy storage systems, the redox chemistry of metal nano-particles embedded in polymer matrices enhances charge storage capacity and cycling stability.^158^ For example, in lithium-ion batteries, the incorporation of silicon nano-particles (SiNPs) into a polymer matrix such as polyacrylonitrile (PAN) improves the anode performance. The chemical reactions during the charge–discharge cycles can be represented as follows:Si + *x*Li + *x*e → Li*x*Si

The chemical environment within the nanocomposite material, including factors such as ionic conductivity and chemical stability, significantly impacts the overall performance of the energy storage device.^139,159^

According to N. S. Shaikh,^160^ a supercapacitor is a great option for a variety of applications since it has a better power density than a battery or fuel cell. For the supercapacitor to work with the battery, its energy density must be increased. To address the low energy density issue, increasing the cell's voltage or capacitance is essential. The energy density (*E*) is proportional to half of the capacitance (*C*) multiplied by the square of the voltage (*V*), as given by formula *E* = ½CV^2^. Choosing appropriate electrode and electrolyte materials can extend the high potential window (cell voltage) and enhance capacitance. Utilizing high porosity carbon electrode materials with increased surface area can also elevate the capacitance of EDLC-type supercapacitors. Therefore, high surface nano-architecture, using high conductivity, and multi-valance state electrode materials can enhance the pseudo-capacitor's performance. Therefore, by using suitable nanocomposites as the materials for pseudo-capacitors and EDLCs, it is possible to boost the energy density of these mixed supercapacitors. Higher ionic conductivity, the right amount of viscosity, and a wider working potential window are all characteristics of an ideal electrolyte that increase energy density. The focus of this article is on the many combinations of electrode materials and graphene-based electrolytes that can be utilized to achieve high stability, energy density, and power density (Fig. 8b).

Table 7 offers a comprehensive overview of research articles investigating various aspects of polymer–metal nanocomposites for energy storage applications, particularly in batteries. Each article explores different materials and technologies, highlighting their potential advantages, challenges, and future prospects. Topics range from conducting polymers-based nanocomposites for supercapacitors and batteries to the use of ferrocene-based polymers and graphene/polymer nanocomposites as electrode materials. Additionally, innovative approaches such as nanostructured high-surface area electrode materials and multifunctional nanocomposite materials for structural batteries are discussed. The studies underscore the importance of advancing polymer–metal nanocomposites to enhance energy storage devices' performance, addressing critical needs for applications spanning electric vehicles, smart grids, and portable electronics.

**Table 7:** Advancing polymer–metal nanocomposites for enhanced energy storage in batteries

Article title	Summary	Polymer–metal nanocomposites for electrode materials in batteries	Research question	Challenges	Potential solutions	Ref.
A review on conducting polymers-based nanocomposites for energy storage application	Examines conducting polymer-based nanocomposites for supercapacitor and batteries, highlighting their advantages, potential synergistic effects, and areas for improvement in capacitances and exploration of ternary nanocomposite designs	Conductive polymer–metal nanocomposites, leveraging properties of CPs and potential synergies with inorganic compounds, for batteries' electrode materials	In what ways might conducting polymer nanocomposites improve batteries and supercapacitors, two types of energy storage devices?	Conducting polymer-based nanocomposites need improvement in capacitances and exploration of ternary nanocomposite designs for better performance in batteries and supercapacitors	Investigate new conducting polymer formulations and synergies with inorganic compounds to optimize performance, and explore the design of ternary nanocomposites to enhance capacitance	161
Organometallic polymer material for energy storage	Potential usage as cathode materials for lithium-ion (Li-ion), sodium-ion (Na-ion), and all-organic batteries is being investigated for ferrocene-based polymers, particularly poly(ferrocenyl-methylsilane) and its derivatives	Battery cathode materials made of ferrocene-based polymers and their derivatives appear promising	Battery cathode materials may be made from ferrocene-based polymers and their derivatives	The potential of ferrocene-based polymers as cathode materials for lithium-ion, sodium-ion, and all-organic batteries is under investigation, but practical implementation and scalability are not yet fully addressed	Conducting further research to validate the efficiency and scalability of ferrocene-based polymers in real-world battery applications, focusing on optimizing their electrochemical properties	162
Graphene/polymer nanocomposites for energy applications	The manufacture and use of graphene/polymer nanocomposites as electrodes in lithium-ion batteries or supercapacitors are investigated in this study. It explores current developments, obstacles, and opportunities for improving energy applications	Graphene/polymer nanocomposites used as electrodes in lithium-ion batteries	What recent progress, difficulties, and potential are associated with creating and using graphene/polymer nanocomposites for energy purposes?	Challenges include improving the integration and performance of graphene/polymer nanocomposites as electrodes in lithium-ion batteries and supercapacitors	Focus on advancing manufacturing techniques and material designs to enhance the compatibility, stability, and performance of graphene/polymer nanocomposites in energy applications	163
Ultrafast all-polymer paper-based batteries	Introduces an innovative electrode material consisting of cellulose fibers coated with polypyrrole, which offers a high surface area at the nanostructural level. The discussion highlights the challenges linked with conducting polymers in batteries and suggests addressing them through novel nanostructured electrode materials	A new electrode material for batteries, featuring cellulose fibers coated with polypyrrole, offers enhanced surface area due to its novel nanostructured composition	What are the challenges associated with conducting polymers for battery applications and how can they be addressed using novel nanostructured high-surface area electrode materials?	Conducting polymers face challenges in battery applications, including limitations in conductivity, stability, and scalability when used as electrode materials	Developing novel nanostructured electrode materials, like cellulose fibers coated with polypyrrole, to address these limitations by increasing surface area and enhancing material stability	164
Conducting polymers and nanocomposites nanowires for energy devices: a brief review	Examines the use of nanocomposite nanowires and conducting polymers in energy devices, focusing on their use in lithium-ion batteries and supercapacitors	Conducting polymers and nanocomposite nanowires used in energy device application	What uses can conducting polymers and nanocomposite nanowires have in energy-related technologies like lithium-ion batteries and supercapacitors?	Conducting polymers and nanocomposite nanowires have potential in energy devices, but there are challenges in optimizing their performance for specific applications like lithium-ion batteries and supercapacitors	Investigating ways to enhance the electrochemical performance and durability of conducting polymers and nanocomposite nanowires, possibly through innovative material designs and synthesis methods	165
Multifunctional nanocomposite materials for energy storage in structural load paths	Aims to create multifunctional nanocomposite materials that can store electric energy within mechanical load channels by examining the electrochemical capability of several commercial carbon fiber grades meant for structural batteries within polymer nanocomposites	Commercial carbon fiber grades' electrochemical capability for polymer nanocomposite structural batteries	How might multifunctional nanocomposite materials be created for the storage of electric energy in mechanical load channels, with a focus on the use of carbon fibers and polymer electrolytes in structural batteries?	Developing multifunctional nanocomposite materials that can store electric energy while bearing mechanical loads presents challenges, particularly in integrating carbon fibers and polymer electrolytes effectively	Exploring the electrochemical capabilities of various carbon fiber grades and refining polymer electrolyte formulations to create robust, multifunctional nanocomposite materials for structural batteries	166
Polymer nanocomposites for lithium battery applications	Examines the most recent advancements in polymer nanocomposites, their difficulties, creative designs, and potential uses in lithium-based battery components such as electrodes, electrolytes, binders, and separators	Polymer–metal nanocomposites as part of polymer nanocomposites used for electrodes in lithium-based batteries	What are the state-of-the-art technologies of polymer nanocomposites for batteries, along with the technological challenges, innovative designs, and future perspectives?	Advancements in polymer nanocomposites for lithium battery components face challenges in enhancing electrode performance, improving electrolytes, and overcoming material limitations	Focusing on state-of-the-art technologies and innovative designs to improve the performance and durability of polymer nanocomposites in lithium batteries, particularly in electrodes, electrolytes, and separators	167
Nanostructured nanocomposites for high energy batteries and supercapacitors	Focuses on the critical need for nanocomposite electrodes to increase the energy and power storage capabilities of high-power energy storage devices, like Li-ion batteries and supercapacitors. These devices are essential for a variety of applications, including portable electronics, smart grids, and electric cars	Nanocomposites of carbon with metal oxides and conductive polymers for battery electrode materials	In what ways can nanocomposite electrodes improve the energy and power storage capabilities of high-power energy storage devices such as lithium-ion batteries and supercapacitors?	There is a critical need to improve the energy and power storage capabilities of nanocomposite electrodes for high-power energy storage devices like lithium-ion batteries and supercapacitors	Researching and developing advanced nanocomposite electrodes combining carbon, metal oxides, and conductive polymers to significantly boost the storage capacities and power delivery of these energy devices	168

Among other nanocomposites, polymer–metal nanocomposites have been studied and developed to address significant problems with technologies for storing energy, including batteries and supercapacitors. Some advantages of employing nanocomposites and their potential effects on various components of these devices are as follows: polymer nanocomposites play a significant role in supercapacitors, especially when it comes to making conductive polymer electrodes. Nanocomposites are often enhanced with materials such as polypyrrole (PPy), polyaniline, and poly(3,4-ethylenedioxythiophene) (PEDOT) to increase their electrical conductivity and charge storage capacity. Polymer nanocomposite electrolytes provide supercapacitors with improved ion conductivity and mechanical durability. The overall stability and performance of the electrolyte are enhanced by the addition of nanomaterials like graphene or carbon nanotubes into polymer matrices. Polymer nanocomposite separator membranes improve mechanical and thermal stability while assisting in the prevention of internal short circuits. For better separator performance, nanofillers like nanocellulose or ceramic nano-particles are frequently added to polymer matrices.^20^

#### 4.4.1. Electrode materials

Batteries and supercapacitors can use polymer–metal nanocomposites as their electrode materials. Combining the advantageous qualities of the metal and polymer components is made possible by the nature of nanocomposites. Polymer–metal nanocomposites can be used as electrode materials in supercapacitors to improve cycling stability, specific capacitance, and rate capability. For instance, the metal component can offer excellent electrical conductivity and electrochemical reactivity, while the polymer can give greater surface area, mechanical stability, and flexibility. Supercapacitors benefit from enhanced mechanical stability and ion conductivity thanks to polymer nanocomposite electrolytes.^159^

#### 4.4.2. Electrolytes and separator materials

Polymer nanocomposite separator membranes improve mechanical and thermal stability while assisting in the prevention of internal short circuits. For better separator performance, nanofillers like nanocellulose or ceramic nano-particles are frequently added to polymer matrices. Nanocomposites are also useful for separator materials and electrolytes, which are essential parts of batteries and supercapacitors. Solid-state electrolytes and separator membranes can benefit from the addition of polymer–metal nanocomposites to promote stability, improve ion transport, and guard against electrode short circuits. These nanocomposite materials offer improved ionic conductivity, mechanical reinforcement, and flexibility.^83^ The overall stability and performance of the electrolyte are improved by the incorporation of nanomaterials such as graphene or carbon nanotubes into polymer matrices.

#### 4.4.3. Performance enhancement

Performance improvements in energy storage systems can be significantly attributed to the incorporation of polymer–metal nanocomposites. These nanocomposites enhance overall energy density, cycling life, and specific capacity of batteries through several mechanisms. For instance, the metal nanoparticles within the polymer matrix provide enhanced electrical conductivity, which facilitates faster electron transport and reduces internal resistance. This results in a higher rate capability and improved efficiency of the energy storage devices.

Additionally, the mechanical flexibility and structural integrity of polymer matrices help mitigate volume changes during charge–discharge cycles, thus enhancing the cycling stability and prolonging the cycling life of the batteries. Specific capacity improvements are achieved through the synergistic effects of polymers and metal particles, which increase the surface area for electrochemical reactions, leading to higher specific capacitance and energy density.

To ensure these materials are suitable for practical applications, rigorous characterization and testing procedures are employed. Techniques such as electrochemical impedance spectroscopy (EIS) and cyclic voltammetry (CV) are used to evaluate the rate capability and efficiency. Scanning electron microscopy (SEM) and transmission electron microscopy (TEM) provide insights into the morphology and dispersion of metal particles within the polymer matrix, which are critical for understanding their impact on cycling stability.^20^

#### 4.4.4. Design flexibility and scalability

Energy storage device manufacturers can benefit from the scalability and design flexibility provided by polymer–metal nanocomposites. Numerous processes, such as printing, coating, and solution casting, can be used to treat them, enabling the creation of complex and unique electrode architecture. Numerous fabrication techniques are affordable and scalable, allowing for the mass production of energy storage devices. Research and development efforts are actively focused on the creation of nanocomposite materials (Fig. 8c), such as polymer–metal nanocomposites, for energy storage devices. Scientists are investigating several approaches to enhance the makeup, structure, and functionality of nanocomposite materials with the goal of achieving elevated energy density, extended longevity, enhanced security, and decreased expenses in supercapacitors and batteries. More attention is being paid to the sustainability and environmental effects of polymer nanocomposite-based supercapacitors and batteries. To make sure that these energy storage options are in line with more general sustainability objectives, researchers are looking into recyclable and environmentally acceptable alternatives for use in the manufacturing process.^83^

To summarize, supercapacitors and batteries are examples of energy storage systems that heavily rely on nanocomposite materials, particularly polymer–metal nanocomposites. They offer benefits such as increased device characteristics, scalability, improved performance, and design flexibility when used as electrode materials, electrolytes, and separator materials. The goal of ongoing research is to further enhance nanocomposite materials for energy storage systems that are sustainable and effective.

### 4.5. Polymer–metal nanocomposites for electronic applications

#### 4.5.1. Flexible and stretchable electronics

The creation of electronics that can stretch, bend, and conform to irregular shapes without losing their function is known as flexible and stretchable electronics. Nanocomposites made of polymers and metals have shown promise as materials for creating stretchy and flexible electronic devices. Since polymer–metal nanocomposites have a special blend of electrical conductivity and mechanical flexibility, they have become a potential material for the construction of these electronic devices.^145^

#### 4.5.2. Material composition and properties

Nanocomposites with remarkable mechanical and electrical qualities are known to be constructed of polymers and conductive metals like copper, gold, or silver. The inherent flexibility of polymers provides the structural foundation, while the addition of metallic components ensures adequate electrical conductivity. This combination enables the creation of tiny, conductive, malleable layers all necessary for electrical applications that are flexible and stretchable.^152^

#### 4.5.3. Fabrication techniques

The creation of functional polymer–metal nanocomposites requires a range of fabrication techniques. Flexible and uniform layers are commonly created using the processes of solution casting, printing, and electrospinning. These methods provide exact control of the nanocomposite's thickness and composition, ensuring optimal performance in electrical applications.^20^

#### 4.5.4. Conformity to irregular shapes

**Fig. 9:**
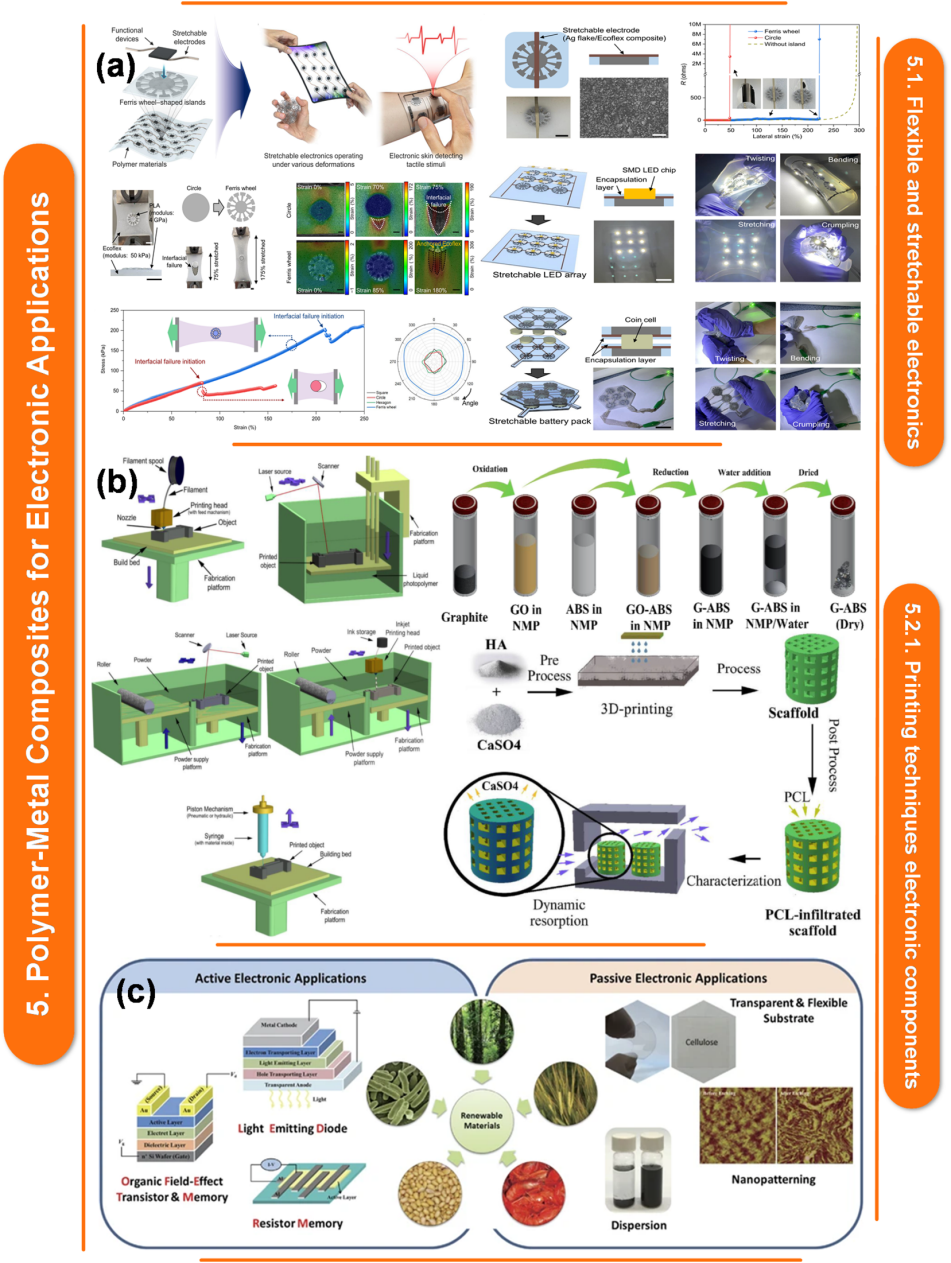
The following are examples of highly durable stretchable electronics: (a) FWIs embedded in an Ecoflex substrate;^179^ this figure has been reproduced from ref. 179 with permission from the American Association for the Advancement of Science (AAAS), copyright 2022. The figure (b) depicts stretchable electronics comprising rigid components capable of enduring various deformation modes.^180^ This figure has been reproduced from ref. 180 with permission from Springer Nature, copyright 2020. (c) AM technology types, such as robocasting, FDM, SLA, SLS, and PLP. Graphene/ABS nanocomposite preparation for 3D printing filament production, PCL-HA-CaSO_4_ scaffold 3D printing technique, diagram showing how renewable polymers are used in electronics.^181^ This figure has been reproduced from ref. 181 with permission from The Society of Polymer Science, Japan, copyright 2017.

Polymer–metal nanocomposites' elasticity makes it easier for them to take on irregular shapes, which is a crucial quality for applications requiring non-conventional form factors. This characteristic is used in applications where conventional rigid electronics would not be feasible, such as conformable sensors, electronic fabrics, and wearable gadgets (Fig. 9a).^172^

#### 4.5.5. Bendability

The bendability of the nanocomposites is an important property that permits electronic components to flex without causing structural damage. This feature is used in the creation of curved sensors, flexible displays, and other gadgets that require a flexible form factor. Flexible electronics must be able to endure bending without losing function in order to be durable and dependable. The chemical bonding and interactions at the polymer–metal interface contribute to the durability and conductivity of flexible electronic materials. Chemical modifications to the polymer matrix, such as doping and functionalization, can further enhance the electrical properties and mechanical flexibility of the nanocomposite.^119^ For instance, doping polypyrrole (PPy) with silver nano-particles (AgNPs) enhances its conductivity:PPy + AgNO_3_ → PPy(Ag) + NO_2_

This doping process introduces conductive pathways within the polymer matrix, improving its suitability for flexible electronic applications.

#### 4.5.6. Stretchability

Stretchability is arguably one of the most unique characteristics of polymer–metal nanocomposites. Electronic devices can stretch and deform thanks to this characteristic, which makes them useful in fields like robotics, healthcare, and electronic skins. These materials' stretchability aids in the production of adaptive and flexible electrical devices that can adjust to dynamic and uneven surfaces.^152^

Soft and conformable electronics are predicted to dominate the next generation of electronic devices. Stretchable conductor design and application were the focus of Lee *et al.*'s study.^172^ They said that these gadgets are becoming more and more popular. Modern electronics cannot function without electrical conductors, which are essential to the development of flexible electronic devices and systems. Materials are essential for producing stretchable conductors with the necessary mechanical and electrical properties. In order to provide high stretchability for applications, elastomeric materials like Ecoflex, polyurethane (PU) and polydimethylsiloxane (PDMS), are often used as the substrate or matrix to interface with other system components. Here are some examples of very durable stretchable electronics: the paragraph explains how stretchy electronics work and how they are used, namely in electronic skin (also known as “e-skin”) to detect tactile stimuli. It describes how these devices are made using advanced manufacturing (AM) processes like PLP, SLA, FDM, SLS, and robocasting. It also talks about how conductive nanomaterials like metallic nanowires and carbon nanotubes are added to improve conductivity (Fig. 9b). It also emphasizes the development of resilient stretchy electronics that can withstand a variety of deformation patterns and the usage of renewable polymers in electronics. Additionally, it discusses the advancement of nanocomposite materials such as ABS/graphene for the production of 3D printing filaments.

#### 4.5.7. Integration of polymer–metal nanocomposites in flexible electronic devices

As shown in Fig. 9c, polymer–metal nanocomposites can be used in flexible electronic devices to offer structural stability, electrical conductivity, and mechanical flexibility. These nanocomposites can be employed as active parts in flexible electronic devices, as well as conductive tracks, electrodes, and interconnects. The polymer component gives the nanocomposite its stretchability and flexibility, which helps it to resist mechanical deformation. Electrical conductivity is provided by the metal component, which can be designed or assembled to form useful circuits. The integration of polymer–metal nanocomposites can enable flexible screens, wearable electronics, flexible sensors, and other flexible electronic components. To achieve the necessary mechanical and electrical characteristics, the structure, composition, and manufacturing methods of polymer–metal nanocomposites used in flexible electronic devices must be optimized. To facilitate the mass manufacture of flexible electronic devices with excellent performance and dependability, researchers are investigating different combinations of metals and polymers as well as fabrication techniques including printing, coating, or deposition.^173^

D. Xu *et al.*^174^ investigated the fascinating field of liquid metal (LM) and its possible uses in elastic and flexible electronics in their paper. Because of its exceptional fluidity and room temperature conductivity, LM has attracted a lot of attention. High surface tension and oxidation susceptibility, however, provide difficulties for material manufacturing and real-world applications. The authors suggest creative ways to get over these obstacles including the composition and nanolization of liquid metal. These methods lessen the impact of high surface tension, improving both processibility and performance. The goal of the review is to present a thorough overview of the methods used in the creation, processing, and use of LM-based nanocomposites. Among these, the composition process is noteworthy for being a potentially effective way to enable printed ink's additional capabilities. The review also explores the latest printing applications of LM-based nanocomposites, providing insightful information and direction for the large-scale manufacturing of stretchable electronics. The chemical bonding and interactions at the polymer–metal interface contribute to the durability and conductivity of flexible electronic materials. Chemical modifications to the polymer matrix, such as doping and functionalization, can further enhance the electrical properties and mechanical flexibility of the nanocomposite.

### 4.6. Printed electronics

#### 4.6.1. Printing techniques for fabricating polymer–metal nanocomposite-based electronic components

For printed electronics applications, printing techniques are essential to the fabrication of polymer–metal nanocomposite-based electronic components. Fig. 9c lists the many AM technologies, such as FDM, SLA, SLS, PLP, and robocasting. The following are some typical printing methods applied in this field:^119^

(i) Fused deposition modelling (FDM): when it comes to 3D printing nanocomposite polymers, FDM is the most popular and fundamental technique. Because thermoplastics melt at lower temperatures, materials like PLA and ABS are typically employed in this process. This process involves feeding specific diameter polymer filaments into the printing head of the printer, where they melt and are extruded onto the base platform layer by layer by the nozzle until they solidify into the final result. The raster angle, air gaps, layer thickness, and orientation are all controlled by the printing settings. Fused deposition modeling has several advantages, including low cost, quick prototyping, and an easy-to-use process. Additionally, two nozzles can be used to print multiple materials at once, creating items with multiple functions.^93^

(ii) Stereolithography (SLA): stereolithography, the process of curing photopolymers with UV laser light, represents the original and fundamental application of liquid photopolymer-based additive manufacturing (AM) technology. In this process, UV laser light, directed by a computer-controlled CAD model, polymerizes and cures the first layer of material. The platform then descends, allowing each subsequent layer to cure and solidify, ultimately forming the desired object. Once printing is finished, any unreacted resin material can be removed and recycled. Post-curing may be employed to achieve specific characteristics and surface finishes. Epoxy and acrylic resins are among the most commonly used polymer compounds in stereolithography. A number of factors, including laser intensity, exposure and curing times, scanning speed, and printing resolution, affect the final product's quality.^113^

(iii) Powder–liquid 3D printing (PLP): this technology is also known as drop-on-powder printing or 3-Dimensional printing (3DP), rooted in powder–liquid processing. Here, powder is spread onto a base platform, and a liquid binder is dispensed onto the powder layer *via* a moving head injector in the *X*–*Y* direction. Once the binder solidifies the coating, the platform descends, and a new layer of powder is added atop the previous one.^132^ This process continues, with the binder continuously injected and the platform lowered, until the final product is created. Once finished, the part's extra powder is taken out and put to good use. As a result of the binder securely binding each layer to the powder-covered substrate, this method creates extremely intricate structures. This approach works well with a variety of materials and needs room temperature to be processed.^175^

(iv) SLS, or selective laser sintering: like powder–liquid printing, it is likewise a powder-based processing method. Rather than fusing the layers together with a liquid adhesive, this approach uses a laser beam. Powder fusion occurs for every layer as a result of the controlled interaction between the powder and the high-power laser beam. Until the finished product, each stage is followed by the piston-operated platform being lowered. The completed portion is cleaned of any leftover powder. The primary factors influencing the high-resolution output are powder size. This technique's primary benefits are its great quality and high resolution. It is a support-free process because the created part's surrounding unbounded powder provides support. However, this method has multiple drawbacks, such as being expensive, time-consuming, and prone to porosity problems when the powder does not melt and fuse correctly.^175^

(v) Casting robots: it's an extrusion-based method also referred to as direct ink writing or 3D-plotting. The material is extruded through a pressured opening while it is typically in a slurry form. In this approach, the product is manufactured layer by layer by the orifice head moving in all three dimensions while the platform remains stationary. Using mixing nozzles to feed two or more reactive materials, or performing a post-thermal/UV light treatment, can improve the curing process. The material viscosity and deposition speed determine the resolution and product quality. There is a wide variety of material flexibility with this technique. As raw materials, ceramics, pastes, suspensions, and solutions of materials can be used. Support is required in the case of intricate constructions and designs because the raw material is soft and has low rigidity.^176^

### 4.7. Sensors and actuators

Polymer–metal nanocomposites, often known as smart materials, have attracted significant interest in the field of sensors and actuators because of their unique characteristics and responsiveness. These nanocomposites usually consist of metallic components embedded in a polymer matrix, resulting in a material that combines the advantageous properties of metals and polymers. The surface chemistry of polymer–metal nanocomposites is critical for their application in sensors. Chemical functionalization of the polymer or metal surface can improve selectivity and sensitivity, allowing the sensor to detect specific analytes with high accuracy and precision. Actuators and sensors are devices that translate environmental or physical stimuli into electrical impulses, and mechanical motion respectively. Since polymer–metal nanocomposites have special qualities and are responsive, they have been investigated for use in sensing and actuation applications. Ionic polymer–metal nanocomposites (IPMCs) have been extensively researched as soft actuators due to their biocompatibility, intrinsic flexibility, and low weight, according to D. Zhao *et al.*^177^ Ionic polymer–metal nanocomposites (IPMCs) exhibit notable deformation at low electrical excitation voltages (usually less than 10 V). Typically, IPMCs are composed of polymer electrolytes sandwiched between noble metal electrodes. These attributes have led to an increased utilization of IPMC actuators in fields such as bionic robotics, biomedicine, and artificial muscles. In water-based IPMC actuators, the migration of water molecules attached to counter-ions to the cathode, driven by an electric field between metal electrodes, contributes to their bending deformation. The presence of water molecules is crucial for IPMC actuation performance. The surface chemistry of polymer–metal nanocomposites is critical for their application in sensors. Chemical functionalization of the polymer or metal surface can improve selectivity and sensitivity, allowing the sensor to detect specific analytes with high accuracy and precision. For example, the functionalization of carbon nanotubes (CNTs) with carboxyl groups (–COOH) enhances their ability to detect ammonia (NH_3_):CNT-COOH + NH_3_ → CNT-CONH_2_ + H_2_O

Unfortunately, there is a water leak in the current IPMC design, which results in substantial attenuation and limited operating durations in ambient air. This study suggests a feasible and efficient way to increase IPMC performance in air, which is to dope polyethylene oxide (PEO) with enhanced water retention capacity into the Nafion membrane. Physical property analysis showed that the PEO/Nafion membrane with a 20-weight percent PEO component had a consistent internal structure and an ideal water uptake ratio. The longest steady working period, biggest peak-to-peak displacement, and maximum volumetric work density were found in IPMCs having 20 weight percent PEO, according to investigations into the electromechanical properties of IPMCs with different PEO levels. These results suggest that doping PEO reduces displacement attenuation and improves the electromechanical characteristics of the resultant IPMC.^178^

#### 4.7.1. Utilization of nanocomposites for sensing and actuation applications

In smart materials research, the use of metal nanocomposites for actuation and sensing applications is a major breakthrough. A soft multiple shape-memory polymer–metal nanocomposite (MSMPMC) actuator with many degrees of freedom was introduced by Q. Shen^176^ and colleagues. The MSMPMC is made up of two or more electrodes spaced apart by an ion-conductive polymer material, just like IPMC actuators. When a voltage is supplied, ions and water molecules move within the polymer due to electrostatic interactions, causing bending deformation, also known as electro-mechanical actuation. This electromechanical actuation effect is inherently gentle, biocompatible, and long-lasting. In conclusion, polymer–metal nanocomposites are essential to printed electronics, flexible and stretchable electronics, sensors, and actuators, among another electron. Their special blend of qualities, such as electrical conductivity, flexibility, and printability, makes them perfect for producing personalized and inventive electronic components. The goal of ongoing research is to optimize polymer–metal nanocomposite composition, fabrication methods, and performance in order to facilitate the creation of next-generation electronic devices with improved functionality and versatility.

## 5. Chemical science of polymer–metal nanocomposites

Polymer–metal nanocomposites exhibit a unique combination of properties due to the intricate chemical interactions between the polymer matrices and embedded metal nanofillers. Understanding these chemical processes is crucial for optimizing the performance of these materials in various applications.

### 5.1. Chemical bonding and interactions

The properties of polymer–metal nanocomposites are significantly influenced by the types of chemical bonds and interactions that occur at the polymer–metal interface. These include:

### 5.2. Covalent and ionic bonds

Covalent and ionic bonds form the backbone of the interactions between polymers and metal nanofillers. These strong chemical bonds are essential for enhancing the mechanical and electrical properties of the nanocomposite materials. For example, consider the interaction between polyaniline (PANI) and gold nanofillers (AuNFs). During the synthesis, gold chloride (AuCl_3_) is reduced *in situ* within the PANI matrix, leading to the formation of gold nanofillers. The reaction can be represented as:PANI + AuCl_3_ → PANI(Au) + 3HClIn this reaction, the gold chloride is reduced to elemental gold, which becomes uniformly distributed within the PANI matrix. This integration significantly enhances the nanocomposite's mechanical strength and electrical conductivity. The covalent bonding between the PANI and the gold nanofillers ensures a strong interface, which is critical for the durability and performance of the nanocomposite.^98^

### 5.3. Van der Waals forces

Although weaker than covalent and ionic bonds, van der Waals forces are crucial for the stabilization of the nanocomposite structure. These forces are responsible for the intermolecular attractions between the polymer matrix and the metal nanofillers. Van der Waals forces play a pivotal role in maintaining the dispersion of metal nanofillers within the polymer matrix. Proper dispersion prevents the aggregation of nanofillers, ensuring uniform properties throughout the nanocomposite. Aggregation can lead to defects and weak points within the material, which can compromise its mechanical and electrical performance. Therefore, even though van der Waals forces are relatively weak, they are essential for achieving a stable and homogeneous nanocomposite structure.^102^

### 5.4. Hydrogen bonding

Hydrogen bonding occurs when polymers contain functional groups capable of forming hydrogen bonds, such as hydroxyl (–OH) or amine (–NH_2_) groups. These bonds significantly influence the stability and properties of the nanocomposite. For instance, in a polymer matrix containing poly(vinyl alcohol) (PVA), hydrogen bonds can form between the hydroxyl groups of PVA and metal hydroxides. This interaction enhances the thermal stability of the nanocomposite material by creating a more robust network of interactions within the matrix. Hydrogen bonding can also improve the mechanical properties of the nanocomposite by increasing the intermolecular forces between the polymer chains and the nanofillers.^108^

### 5.5. Surface interactions

The surface chemistry of metal nanofillers and their interaction with the polymer matrix are critical determinants of the nanocomposite's electrical and thermal properties. Surface interactions involve the modification of the nanofillers to improve their compatibility with the polymer matrix. Surface functionalization of metal nanofillers, such as coating with organic ligands, can significantly enhance their dispersion and integration within the polymer. For example, silica nanofillers can be functionalized with silane coupling agents, which improve their compatibility with polyethylene terephthalate (PET) matrices. This modification ensures better dispersion of the nanofillers and enhances the nanocomposite's mechanical and thermal properties. Furthermore, surface interactions are crucial for the formation of a stable interface between the polymer and the metal nanofillers. A stable interface prevents delamination and ensures that the mechanical and electrical properties of the nanocomposite are maintained under various conditions. Strong surface interactions also contribute to the overall durability and longevity of the nanocomposite material.^123^

In addition, the chemical interactions and processes involved in the fabrication and application of polymer–metal nanocomposites are critical for their performance. Covalent and ionic bonds, van der Waals forces, hydrogen bonding, and surface interactions all play significant roles in determining the properties of these materials. A deep understanding of these chemical aspects can lead to significant advancements in the development of polymer–metal nanocomposites for a wide range of technological applications, including energy storage systems, flexible electronics, and sensors. By optimizing these chemical interactions, it is possible to enhance the performance, stability, and durability of polymer–metal nanocomposites, making them suitable for various advanced applications.

## 6. Advanced applications and future prospects

**Fig. 10:**
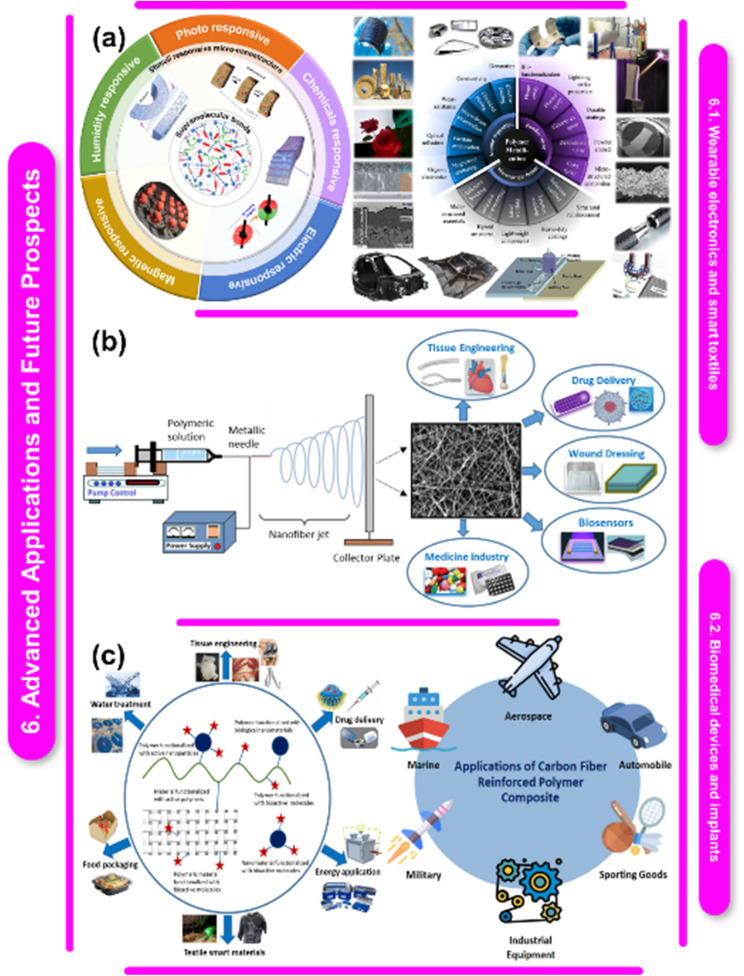
Advanced applications and future prospects of metal–polymer nanocomposites (a–c).^182–184^ This figure has been reproduced from ref. 182–184 with permission from Springer Nature Singapore (for ref. 182), Elsevier (for ref. 183), and MDPI (for ref. 184), copyright 2022 (for ref. 182), 2022 (for ref. 183), and 2023 (for ref. 184).

The unique combination of qualities coming from both metal and polymer constituents has attracted great interest in metal–polymer nanocomposites. These materials are used in many different industries, and continued study points to exciting developments and opportunities, as shown in Fig. 10. Some cutting-edge uses and future directions for metal–polymer nanocomposites include the following:

### 6.1. Wearable electronics and smart textiles

Wearable electronics and smart textile development are greatly influenced by polymer–metal nanocomposites (Fig. 10a). These nanocomposites make it possible to incorporate electrical features into apparel and accessories, resulting in flexible, comfortable, and multipurpose wearable technology. Polymer–metal nanocomposites can be used to create fabrics with conductive tracks, sensors, and actuators completely incorporated. This enables the use of haptic feedback systems, gesture recognition, and real-time physiological parameter monitoring. These nanocomposites' stretchability and flexibility guarantee that they are compatible with the dynamic movements of the human body. The creation of smart textiles with responsive qualities, such as thermochromic or electrochromic behavior, which can alter color or transparency in response to external stimuli, is also made possible by polymer–metal nanocomposites. Fashion, athletics, healthcare, and other fields where interactive and adaptable apparel is sought find uses for these smart textiles.^182^

### 6.2. Biomedical devices and implants

Polymer–metal nanocomposites are very promising for implants and biomedical devices (Fig. 10b). The development of implanted devices that can monitor, stimulate, or repair biological functions is made possible by the combination of conductive metals and biocompatible polymers. Electrodes for neural interfaces made of polymer–metal nanocomposites can facilitate communication between electronic equipment and the nervous system. These nanocomposites can potentially be used in therapeutic devices for targeted medication delivery or in biosensing platforms for ongoing physiological parameter monitoring. Biodegradable polymer–metal nanocomposites have also made it easier to create temporary implantable implants that eventually break down, minimizing the need.^183^

### 6.3. Internet of things (IoT) applications

An important factor in the development of Internet of Things (IoT) applications is the use of polymer–metal nanocomposites (Fig. 10c). The production of adaptable and lightweight sensors, energy harvesters, and communication components depends heavily on these nanocomposites. Polymer–metal nanocomposites can be used to construct flexible and conformable sensors that measure physical qualities, detect gasses, and monitor environmental parameters in internet of things devices. These nanocomposites are used in energy harvesters, which effectively transform ambient energy such as temperature gradients or vibrations into electrical energy for IoT device power. Furthermore, responsive and dependable wireless communication within the internet of things networks is ensured by the incorporation of polymer–metal nanocomposites in communication components like antennas and RFID tags.^184^

### 6.4. Emerging applications and future trends

Beyond the current applications, polymer–metal nanocomposites are anticipated to play a transformative role in several emerging fields. For instance, in the realm of quantum computing, these materials could be used to create advanced qubits or components for quantum circuits, thanks to their unique electrical and magnetic properties. Additionally, in the field of environmental remediation, polymer–metal nanocomposites could be engineered to create filters or catalytic surfaces that can remove pollutants from air or water with unprecedented efficiency. Another promising area is in the development of next-generation batteries and supercapacitors. Polymer–metal nanocomposites could be key in creating more efficient and durable energy storage devices, particularly those that are flexible and lightweight, which would be essential for powering portable electronics and electric vehicles.

Finally, the integration of polymer–metal nanocomposites in additive manufacturing and 4D printing could lead to the production of smart materials and structures that can change shape, properties, or function over time, in response to external stimuli. This capability could have profound implications for sectors ranging from aerospace to medical devices, enabling the creation of materials and devices that can adapt to their environment or usage conditions in real-time.

### 6.5. Emerging trends and future directions

**Fig. 11:**
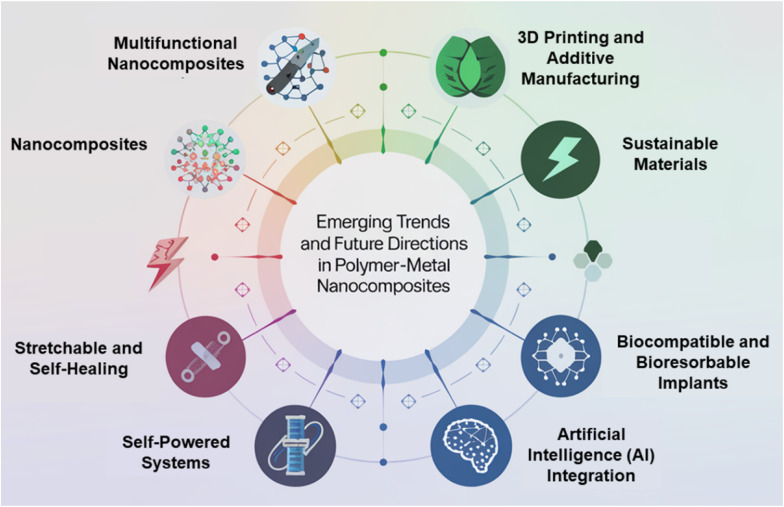
This figure illustrates the emerging trends and future directions in polymer–metal nanocomposites, with the central theme highlighting the core focus of the field. Surrounding it are key areas of development: multifunctional nanocomposites, 3D printing and additive manufacturing, sustainable materials, biocompatible and bioresorbable implants, AI integration, self-powered systems, and stretchable, self-healing materials. Each trend is represented by an icon, symbolizing its unique characteristics and potential impact on the future of materials science and technology.

Many new trends and potential paths are being investigated in the field of polymer–metal nanocomposites (Fig. 11):

(a) Multifunctional nanocomposites: scientists are working on creating polymer–metal nanocomposites that have several uses, like antibacterial activity, self-cleaning surfaces, or self-healing capabilities. The goal of these multipurpose nanocomposites is to improve the materials' overall durability and performance in a range of applications.

(b) Nanocomposites: there is an ongoing study on the integration of metal nanostructures, such as nanowires or nano-particles, into polymer matrices. Better mechanical, electrical, and optical qualities provided by nanocomposites enable higher performance in a variety of applications, from energy storage to electronics.

(c) Hybrid nanocomposites: in order to attain synergistic features and broaden the spectrum of applications, hybrid nanocomposites which blend polymer–metal nanocomposites with other materials, such as ceramics or carbon-based materials are being investigated. The mechanical strength, electrical conductivity, or thermal stability of these hybrid nanocomposites are improved.

(d) Sustainable materials: creating sustainable polymer–metal nanocomposites through the use of renewable polymers, environmentally friendly synthesis techniques, and recycling strategies is becoming more and more important. The goal is to preserve these materials' functionality and performance while lessening their negative effects on the environment.

(e) Self-powered systems: research is being done on polymer–metal nanocomposites in order to create self-powered systems that can gather and store energy to run electronic gadgets on their own. To develop self-sustaining power sources, these systems combine energy harvesting elements such as piezoelectric or thermoelectric element technologies for storing energy, such as batteries or supercapacitors.

(f) Materials that are stretchable and self-healing: scientists are trying to create polymer–metal nanocomposites with these qualities. These materials possess the capacity to self-repair in the event of minor damage and can tolerate significant deformations without causing structural harm. Nanocomposites that are stretchable and self-healing may find use in electronic skins, flexible displays, and wearable electronics.

(g) Biocompatible and bioresorbable implants: the field of biomedical implants is paying more attention to the development of biocompatible and bioresorbable polymer–metal nanocomposites. During the healing process, these nanocomposites can offer brief electrical stimulation or assistance. Over time, they will gradually break down and be absorbed by the body. This method enhances patient comfort and recuperation while lowering the necessity for implant removal procedures.

(h) 3D Printing and additive manufacturing: by combining polymer–metal nanocomposites with these technologies, new avenues for the creation of intricate structures and useful gadgets are opened up. Better-performing electronic components and individualized, patient-specific implants can be made possible by the exact control of the composition and geographical distribution of nanocomposite materials.

(i) Artificial intelligence (AI) integration: research on the combination of polymer–metal nanocomposites and AI technologies, like machine learning and neural networks, is just getting started. It is conceivable to create intelligent systems that can evaluate and comprehend complex data, make judgments, and modify their behavior by fusing AI algorithms with the nanocomposites' sensing capabilities.

Moreover, Table 8 summarizes the key differences between this review and other similar reviews on polymer–metal nanocomposite fabrication techniques. This review stands out by focusing on the latest advanced techniques like electrohydrodynamic (EHD) processing, aerosol jet printing, and the integration of machine learning, while addressing specific challenges such as scalability, environmental sensitivity, and ink stability. It highlights innovative applications in energy storage, flexible electronics, and sensors, and proposes forward-thinking future research directions, including hybrid techniques and enhanced process control. In contrast, other reviews tend to focus on traditional methods, discuss more general limitations, and suggest incremental improvements rather than exploring groundbreaking new approaches.

**Table 8:** Comparison of the this review with other review articles on polymer–metal nanocomposites

Aspect	This review	Other reviews	Ref.
Key insights	This review delves into the latest and most advanced fabrication techniques, such as electrohydrodynamic (EHD) processing, aerosol jet printing, and the integration of machine learning in fabrication processes. These cutting-edge approaches distinguish this work	Other reviews tend to focus on more traditional methods like CVD, PVD, electrodeposition, and electrospinning, often without detailed exploration of newer, innovative techniques	185–203
Identified limitations	Specific challenges are addressed, such as the scalability of advanced techniques, sensitivity to environmental factors, and issues like ink stability in aerosol jet printing	Most other reviews mention general limitations related to synthesis and characterization but do not delve deeply into the modern-day challenges of newer techniques	
Challenges discussed	This review highlights the practical difficulties of implementing state-of-the-art techniques, including nozzle clogging in EHD and the complexity of achieving uniform ink deposition with aerosol jet printing	Other reviews often focus on traditional challenges, such as achieving even nanoparticle dispersion in polymers and consistent material uniformity	
Applications highlighted	The review emphasizes exciting new applications, particularly in energy storage, flexible electronics, and advanced sensors, focusing on how these techniques can be transformative in these fields	Other reviews typically discuss well-established applications, such as in catalysis, antimicrobial treatments, and basic electronic devices, often without covering the most recent innovations	
Future research directions	Forward-thinking ideas are proposed, including exploring hybrid fabrication techniques, improving ink formulations, and using machine learning to better control fabrication processes	Many other reviews suggest refining existing methods or making incremental improvements, but they do not push the boundaries as much in terms of proposing bold new research directions	

All things considered, the future of polymer–metal nanocomposites entails constant progress in materials science, processing methods, and application-specific customization. Electronics, healthcare, energy, and other industries could all undergo radical change because of these nanocomposites. In order to further improve the characteristics, uses, and sustainability of polymer–metal nanocomposites, research and development activities are now underway. This will open the door for innovative breakthroughs in the years to come.

## 7. Challenges, future directions, and potential solutions

### 7.1. Scalability and manufacturing considerations

Challenges: one of the key obstacles to the widespread adoption of polymer–metal nanocomposites is ensuring that they can be produced on a large scale in a cost-effective manner. As new applications are discovered, it's essential to develop manufacturing processes that can produce these materials in high volumes without sacrificing quality or performance. This challenge involves optimizing high-throughput production techniques like electrodeposition, inkjet printing, and 3D printing.

Potential solutions: to address these challenges, research should focus on improving existing fabrication techniques to increase production speed while maintaining high material quality. This might include enhancing electrodeposition for more uniform coatings, refining inkjet printing for precise material deposition, and advancing 3D printing for creating complex structures. Additionally, combining different manufacturing processes could provide a pathway to more scalable and cost-effective production.

### 7.2. Stability and reliability of polymer–metal nanocomposite films

Challenges: the long-term stability and reliability of polymer–metal nanocomposite films are crucial for their performance, especially in challenging environments. Over time, these materials can face issues like oxidation, delamination, or degradation, particularly when exposed to mechanical stress or harsh conditions.

Potential solutions: future research should prioritize the development of protective coatings that can shield nanocomposite films from environmental damage. Improving the bonding between polymer and metal components could also reduce the risk of delamination. Incorporating more stable and corrosion-resistant metals into the nanocomposite design could further enhance the durability of these materials.

### 7.3. Integration with existing technologies

Challenges: successfully integrating polymer–metal nanocomposites into existing technologies requires overcoming compatibility and interface issues. This includes ensuring that these materials can work with traditional fabrication processes like additive manufacturing for complex structures or microfabrication for electronic devices.

Potential solutions: to facilitate integration, research should focus on modifying polymer–metal nanocomposite formulations to improve compatibility with current fabrication techniques. Tailoring the mechanical, thermal, and electrical properties of these materials can help ensure they interact seamlessly with other components. Developing standardized protocols for incorporating these nanocomposites into various manufacturing processes could also streamline their adoption.

### 7.4. Environmentally friendly approaches

Challenges: with growing emphasis on sustainability, there is a need to develop more environmentally friendly methods for the synthesis, processing, and disposal of polymer–metal nanocomposites.

Potential solutions: research should explore the use of recyclable or biodegradable polymers in creating polymer–metal nanocomposites, along with adopting green synthesis techniques that minimize hazardous chemicals and reduce energy consumption. Efforts should also focus on reducing waste during manufacturing and developing strategies for recycling or safely disposing these materials at the end of their life cycle.

In conclusion, while polymer–metal nanocomposites hold great promise across various applications, several challenges must be addressed to achieve widespread adoption. By focusing on improving production processes, enhancing stability and reliability, ensuring compatibility with existing technologies, and embracing sustainable practices, the continued development and application of these materials will advance the field of materials science and technology.

## 8. Conclusion

This review has explored both conventional and cutting-edge fabrication techniques for polymer–metal nanocomposite films. By highlighting the latest developments, like nanolithography, advanced laser-assisted deposition, and the integration of machine learning, this review sets itself apart from existing studies and outlines a path for future research. These innovations hold great promise for expanding the use of polymer–metal nanocomposites in areas like energy storage and flexible electronics. Moving forward, it's essential for research to keep pushing the boundaries of these advanced techniques to unlock the full potential of these versatile materials.

### 8.1. Suggestions for future research and development

Future studies and development initiatives in the realm of polymer–metal nanocomposites should concentrate on a number of important areas. It is first necessary to investigate scalable and economical manufacturing methods in order to produce these nanocomposites in large quantities without sacrificing their quality and performance. To guarantee the long-term operation of polymer–metal nanocomposite films, stability and reliability challenges need to be solved, especially in harsh environmental circumstances. In order to increase compatibility and interface interactions, integration with current technologies and materials should be maximized. Moreover, during the whole life cycle of these nanocomposites, ecologically friendly and sustainable techniques including the usage of biodegradable polymers and green synthesis techniques should be taken into account. In order to improve the characteristics and uses of polymer–metal nanocomposites, more study should be done on cutting-edge developments such as hybrid nanocomposites, nanocomposites, and multifunctional nanocomposites. Exciting developments in the subject include the creation of biocompatible and bioresorbable implants, flexible and self-healing materials, and self-powered systems. Utilizing 3D printing and additive manufacturing processes along with artificial intelligence (AI) opens up new possibilities for personalization and innovation.

Finally, polymer–metal nanocomposite films have shown to be a flexible and promising material with uses in the biomedical, electronics, and energy sectors. These nanocomposites are anticipated to continue making major contributions to a number of industries through continued research and development, problem-solving, and exploration of future directions. These developments will likely create new avenues for the development of advanced electronic devices, more effective energy use, and enhanced healthcare technologies.

## Data availability

No primary research results, software or code has been included and no new data were generated or analysed as part of this review.

## Author contributions

Muhammad Tayyab: lead author, contributed to the conceptualization, research, and writing of the manuscript. Sajid Rauf: provided expertise in electrochemical processes and contributed to the sections on electrodeposition and electrospinning. Liu Zizhe: assisted in literature review and analysis of chemical vapor deposition techniques. Zixuan Xu: contributed to the research on physical vapor deposition and the optimization of material properties. Rizwan Ur Rehman Sagar: provided insights into the applications of polymer–metal nanocomposites in energy storage systems. Zuhra Tayyab: assisted in data collection and analysis, particularly in the field of flexible electronics. Rashid Ur Rehman: contributed to the section on surface interactions and chemical bonding at the polymer–metal interface. Muhammad Imran: provided technical expertise in the fabrication techniques discussed in the review. Anjam Waheed: assisted in the sustainability aspects of the review, focusing on green chemistry principles. Faisal Faiz: contributed to the research on the applications of polymer–metal nanocomposites in sensors. Rida Javed: assisted in the writing and editing of the manuscript, focusing on the introduction and conclusion sections. Arunpandiyan Surulinathan: provided critical feedback and contributed to the revisions of the manuscript. Zulakha Zafar: assisted in the review of relevant literature and the synthesis of information. Xian-Zhu Fu: provided overall guidance and oversight of the research process. Jing-Li Luo: corresponding author, provided supervision, critical review, and final approval of the manuscript.

## Conflicts of interest

There are no conflicts to declare.
