# Correction: Advanced fabrication techniques for polymer–metal nanocomposite films: state-of-the-art innovations in energy and electronic applications

**DOI:** 10.1039/d5sc90087e

**Published:** 2025-04-25

**Authors:** Muhammad Tayyab, Liu Zizhe, Sajid Rauf, Zixuan Xu, R. U. R. Sagar, Faisal Faiz, Zuhra Tayyab, Rashid Ur Rehman, Muhammad Imran, Anjam Waheed, Rida Javed, A. Surulinathan, Zulakha Zafar, Xian-Zhu Fu, Jing-Li Luo

**Affiliations:** a College of Civil and Transportation Engineering, Shenzhen Key Laboratory of Energy Electrocatalytic Materials, Guangdong Provincial Key Laboratory of New Energy Materials Service Safety China; b College of Materials Science and Engineering, Shenzhen University Shenzhen 518055 China xz.fu@szu.edu.cn jingli.luo@ualberta.ca jll@szu.edu.cn; c College of Mechatronics and Control Engineering, Shenzhen University Shenzhen 518000 China; d Institute for Frontier Materials, Deakin University Waurn Ponds Victoria Australia; e College of Electronic and Information Engineering, Shenzhen University Shenzhen China; f Graduate School of Engineering, Tottori University Tottori Japan; g School of Civil and Environmental Engineering, Nanyang Technological University 639798 Singapore

## Abstract

Correction for ‘Advanced fabrication techniques for polymer–metal nanocomposite films: state-of-the-art innovations in energy and electronic applications’ by Muhammad Tayyab *et al.*, *Chem. Sci.*, 2025, **16**, 3362–3407, https://doi.org/10.1039/D4SC04600E.

The authors regret that the affiliations of the authors with affiliations (a,b) were given incorrectly in the initial publication. The corrected list of affiliations is given below.

Muhammad Tayyab, Liu Zizhe, and Sajid Rauf were also listed incorrectly as equally contributing first authors. Muhammed Tayyab is the sole first author of this paper.

The Royal Society of Chemistry apologises for these errors and any consequent inconvenience to authors and readers.

